# Novel Neuroprotective Potential of *Bunchosia armeniaca* (Cav.) DC against Lipopolysaccharide Induced Alzheimer’s Disease in Mice

**DOI:** 10.3390/plants11141792

**Published:** 2022-07-07

**Authors:** Haidy A. Abbas, Ahmed M. Salama, Sayed A. El-Toumy, Abeer A. A. Salama, Soad H. Tadros, Rania A. El Gedaily

**Affiliations:** 1 Department of Pharmacognosy, Faculty of Pharmacy, Ahram Canadian University, Giza 12573, Egypt; dr.haidyashraf@gmail.com (H.A.A.); am_salama@hotmail.com (A.M.S.); 2 Chemistry of Tannins Department, National Research Centre, El Buhouth St., Dokki, Cairo 12622, Egypt; sayedeltomy@yahoo.com; 3 Department of Pharmacology, National Research Centre, El Buhouth St., Dokki, Cairo 12622, Egypt; berrotec@yahoo.com; 4 Department of Pharmacognosy, Faculty of Pharmacy, Cairo University, Kasr El-Aini St., Cairo 11562, Egypt; soad.tadros@pharma.cu.edu.eg

**Keywords:** Malpighiaceae, *Bunchosia armeniaca*, Q-TOF LC/MS/MS, antioxidant, Alzheimer’s disease, IL-1β, neuroprotective, CYP2E1, flavonoids

## Abstract

*Bunchosia armeniaca* (Cav.) DC (Malpighiaceae) is one of the well-known traditionally used remedies worldwide. This study aims to explore the leaves’ metabolome via Quadrupole-Time-of-Flight-Liquid-Chromatography-Mass Spectrometry and to investigate the neuroprotective effect of leaves using lipopolysaccharide (LPS) induced Alzheimer’s disease model. Mice were administered LPS (0.25 mg/kg/day; intraperitoneal) as well as methanolic extract (BME), dichloromethane (BDMF), and butanol (BBF) fractions (each 200 mg/kg/day; oral) for one week. BME and BBF improved behavioral activity on the Y maze test, decreased brain content of inflammatory markers such as nuclear factor kappa B and interleukin 1 beta, and prevented the elevation of cytochrome P450 2E1, and glial fibrillary acidic protein compared to the LPS-administered group. Histopathological examination of several brain parts confirmed the neuroprotective effect of the tested extracts. In addition, BBF exhibited higher activity in all tested in vitro antioxidant and acetylcholinesterase inhibition assays. Metabolic profiling offered tentative identification of 88 metabolites, including mainly flavonoids, phenolic acids, and coumarins. Several detected metabolites, such as quercetin, apigenin, baicalin, vitexin, and resveratrol, had previously known neuroprotective effects. The current study highlighted the possible novel potential of *B. armeniaca* in preventing memory impairment, possibly through its antioxidant effect and inhibition of acetylcholinesterase, inflammatory and oxidative stress mediators.

## 1. Introduction

The genus *Bunchosia*, family Malpighiaceae, includes approximately 75 species native to America and found mainly in Amazonia, Atlantic Forest, and Pantanal of Brazil [1,2]. *Bunchosia armeniaca* (Cav.) DC. is native to Colombia, Ecuador, Peru, and Bolivia in the Andean region. It is a rare plant in Brazil, although it is widely grown in other South American countries [3]. The genus name derives from one of the Arabic names, “*buncho*”, for the coffee plant (*Coffea arabica* L.) because of the resemblance of the seeds. In contrast, the species name refers to the shape and color of the fruits, such as apricots (*Prunus armeniaca* L.) [4]. In 1789, it was given the name *Malpighia armeniaca*, and renamed *Bunchosia armeniaca* in 1824. This species’ seeds, or pits, are poisonous, although the fruits are edible. The softened red fruits have a strawberry jam taste and a peanut butter consistency [5]. This plant has been utilized in traditional medicine to treat endocrine, infectious, inflammatory, nutritional, and metabolic disorders as well as cancer diseases [6]. Despite the large number of species in the Malpighiaceae family, only a few have been investigated for their chemical aspects. The phytochemical analysis of *B. armeniaca* revealed the presence of several bio-active compounds possessing wide bioactivities such as antimicrobial, antibacterial, antioxidant, and antiinflammation activities [1,7,8]. The only reported flavonoids from this plant’s leaves were rutin, afzelin, isoquercitrin, kaempferol, and quercetin [8].

Natural polyphenols, a large category of substances found in plant-based foods, protect against cancer, cardiovascular, metabolic, and neurological disorders [9,10,11,12]. Polyphenols have drawn much interest as potential therapeutic agents in neurodegenerative illnesses due to well-documented evidence of their antioxidant properties in vitro and in vivo, particularly in Alzheimer’s disease (AD) [13].

AD is a neurodegenerative disease that primarily influences the elderly and results in behavioral changes [14]. Millions of people worldwide are affected by AD, for which only symptomatic treatments are now available. Nowadays, roughly 6.5 million patients aged 65 or older are living with AD in the United States. This number could rise to 13.8 million by 2060, unless medical breakthroughs to prevent, halt, or cure AD are developed. AD was officially reported as the sixth-main cause of mortality in the United States in 2019, and the seventh in 2020 and 2021 [15]. Cognitive and mental functions deteriorate slowly, gradually, and irreversibly in AD. Additionally, the disorder causes significant memory loss as well as an inability to develop new memories, resulting in behavioral problems. AD is regarded as a multi-factorial disorder due to a combination of genetic and environmental variables [16]. There are seven opposing ideas linked to the start of AD: changes in the cholinergic system [17,18]; deposition of beta-amyloid aggregates caused by the incorrect breakdown of the precursor protein of amyloid (APP) [19]; hyperphosphorylation of tau proteins causing the formation of intracellular neurofibrillary tangles [20]; oxidative stress [21,22]; chronic peripheral [21,23] and neuro-inflammation [24] caused by microglial activation; high heavy metal concentrations [25], and metabolic disorders such as dysregulation of cholesterol homeostasis [26], type 2 diabetes, and obesity [27].

Lipopolysaccharide (LPS) is a crucial cell wall constituent of *Gram*-negative bacteria that acts as a potent activator of neuroinflammation, which induces neurodegeneration [28]. Many investigations employed LPS, including in vitro and in vivo amyloidosis and neuroinflammation studies. LPS-induced systemic inflammation has been used to imitate a range of neurodegenerative disorders, including AD, Parkinson’s disease, amyotrophic lateral sclerosis, and multiple sclerosis [29]. Sickness behaviors are a broad category of non-specific behavioral consequences caused by LPS. Reduced activity, decreased exploration, decreased social contact, fever, reduced food and drink consumption, hypersomnia, stimulation of the hypothalamic–pituitary–adrenal (HPA) axis, and enhanced sympathetic activation are examples of these behaviors [30].

The current study aimed to investigate the neuroprotective effects of *B. armeniaca* leaves methanolic extract (BME), as well as, dichloromethane (BDMF), and butanol (BBF) fractions in the management of LPS-induced AD. The behavioral activity on the Y maze was studied to reveal its possible learning and memory-enhancing potential. Brain content of inflammatory markers such as nuclear factor kappa B (NF-κB) and interleukin 1 beta (IL-1β), as well as cytochrome P450 2E1 (CYP2E1), and glial fibrillary acidic protein (GFAP) were assessed, and histopathological features in the brain tissue were examined. Antioxidant assays by 2,2-diphenyl-1-picryl-hydrazyl (DPPH), 2,2-azino bis (3-ethylbenzothiazoline-6-sulphonic acid) (ABTS), and ferric reducing antioxidant power (FRAP) as well as anticholinesterase activity were also performed. Furthermore, metabolites of *B. armeniaca* leaves were analyzed using Quadrupole-Time-of-Flight-Liquid-Chromatography-Mass Spectrometry (Q-TOF LC/MS/MS) to identify constituents that may be relevant to the plant’s neuroprotective action.

## 2. Results and Discussion

### 2.1. Quantitative Determination of Phenolics and Flavonoids

Secondary metabolites, particularly phenolic acids and flavonoids have been previously recognized to treat various disorders and have essential pharmacological effects [31]. The current results revealed that total phenolic (TPC) and flavonoid (TFC) contents in the leaves, expressed as gallic acid and rutin equivalents, were 34.43 ± 1.3 and 13.15 ± 1.25 μg/mg extract, respectively. Hence, *Bunchosia armeniaca* leaves showed considerable TPC and TFC. According to previous research, the total phenolic content of *B. armeniaca* fruit was calculated to be 870.80 ± 8.28 mg gallic acid equivalent per 100 gm of fruit extract [7]. Moreover, the content of flavonoids in *Bunchosia glandulifera* fruit at different stages of ripening was previously reported to be 63.46 ± 5.06, 73.24 ± 4.89, 85.65 ± 8.37, and 98.34 ± 6.28 mg quercetin equivalent/100 g in green, yellow, orange, and red, respectively. During the ripening process, the level of flavonoids in the fruit increased significantly. This could be due to the carotenoid synthesis during the color transition from green to yellow, which is correlated to increased flavonoid quantity [32]. The difference in total phenolic and flavonoid content values may occur due to the difference in plant parts studied.

### 2.2. Metabolic Profiling Using Q-TOF LC/MS/MS

LC–MS approaches are anticipated to be of exceptional utility in plants. They have a reasonably rich biochemistry that comprises several semi-polar components, including crucial secondary metabolite classes, that may be efficiently separated and identified using LC–MS techniques [33]. Electrospray ionization (ESI), in both positive and negative ion modes, has swiftly become the method of choice for sugar sequence analysis, especially when combined with MS–MS techniques. The approach helps use minimal quantities of constituents (less than 1 mg) and facilitates rapid analysis of pure and mixed samples with no need for derivatization [34].

Metabolomic analysis of BME leaves using Q-TOF LC/MS/MS was adopted to unravel the phytoconstituents. Negative and positive modes were performed in the current study, leading to the tentative identification of 88 compounds from distinct phytochemical classes in *B. armeniaca* leaves (Table 1). The identified compounds included 13 amino acids, 3 alkaloids, 6 phenolic acids, 36 flavonoids, 4 coumarin, 9 polyphenols, and 17 miscellaneous, arranged according to their retention time (RT). Base peak chromatograms (BPC) in positive and negative ionization modes are shown in [Figure 1, Figure 2, Figures S1 and S2]. The characteristic fragmentation patterns of some identified compounds on the basis of ESI-MS/MS data are presented in Figures S3–S24.

#### 2.2.1. Flavonoids

There were 36 peaks assigned to flavonoids, all of which belonged to various subclasses: flavonols, flavones, flavanonols, isoflavones, and flavanones. Eight flavone glycosides were characterized (peaks: 32, 38, 44, 51, 52, 54, 57, and 63), in addition to twelve flavonol glycosides (peaks: 34, 35, 39, 40, 42, 43, 47, 53, 56, 62, 65, and 81), two isoflavone glycosides (peaks 50 and 60), three flavanone glycosides (peaks 59, 61, and 70), three flavone aglycone (peaks 66, 69, and 79), one isoflavone aglycone (peak 74), five flavanol aglycone (peaks 46, 75, 76, 83, and 84), one flavanone aglycone (peak 80), and one flavanonol aglycone (peak 41). The A and B-ring mass fragments and small molecule fragments were utilized as structure elucidation markers for the flavonoid aglycone, with 18 Dalton (Da) for H_2_O, 28 Da for C=O, and 42 Da for CH_2_C=O [35]. The sugar residues glucose, rhamnose, and rutinose vanished in the flavonoid glycosides, with a loss of 162, 146, and 308 Da, respectively [36].

##### Flavonoid Triglycosides

One flavonoid triglycoside was detected in *B. armeniaca* corresponding to kaempferol-3-*O*-robinoside-7-*O*-rhamnoside (peak 47). It displayed a molecular ion peak at *m*/*z* 741.2262 and distinctive fragment ions at *m*/*z* 595.1675 [(M + H)-146] and 433.1141 [(M + H)-308] due to the loss of rhamnose and robinose units, respectively [37].

##### Flavonoid Diglycosides

In total, eight flavonoid diglycosides were characterized in the studied *B. armeniaca* extract. Compound 34 was tentatively identified as kaempferol-7-*O*-neohesperidoside. It displayed a precursor ion peak at *m*/*z* 593.1525 with a characteristic product ion at *m*/*z* 285.0508 due to loss of neohesperidosyl moiety (−308 Da) [38]. Luteolin-3′, 7-di-*O*-glucoside (peak 57) was detected at *m*/*z* 609.1534, with a product ion peak at *m*/*z* 285.0479, related to free luteolin as a result of the loss of two hexosyl moieties [39]. Compound 59 (eriodictyol-7-*O*-neohesperidoside) had a pseudo molecular ion at *m*/*z* 595.2007 with a main fragment peak *m*/*z* 287.1189 after loss of a hexosyl moiety (−162 Da) and a deoxy hexosyl moiety (−146 Da) [40]. Furthermore, peaks 38, 52, and 65 displayed molecular ion peaks at *m*/*z* 593.2739, 609.1923, and 625.1824, with fragment ion peaks of the aglycone moieties at *m*/z 285.1368, 301.2999, and 317.0589, respectively, correlating to the loss of a rutinose unit. Thus, peaks 38, 52, and 65 were designated as acacetin-7-*O*-rutinoside, diosmetin-7-*O*-rutinoside, and isorhamnetin-3-*O*-rutinoside, respectively [41,42,43]. Compound 61 was tentatively identified as isosakuranetin-7-*O*-neohesperidoside with precursor and product ions at *m*/*z* 595.1644 and 287.0566, respectively. The fragment ion at *m*/*z* 287.0566 was generated by the loss of glucose and rhamnose sugar moieties [(M + H)-308] [44].

##### Flavonoid Monoglycosides

Sixteen flavonoid monoglycosides were tentatively identified in the current study. Three mono-*C*-glycosyl flavones (compounds 51, 54, and 63) produced typical MS fragmentation patterns of *C*-glycoside flavonoids, such as dehydration and cross ring cleavage of the glucose residue, resulting in 0, 2 cross ring cleavage [(M + H)-120] and 0, 3 cross ring cleavage [(M + H)-90] [45]. Compounds 51, 54, and 63 had pseudo molecular ions at *m*/*z* 449.1086, 433.1491, and 449.1419, respectively, and displayed characteristic *C*-glycoside fragmentation patterns. Hence, they were tentatively named luteolin-6-*C*-glucoside (isoorientin), apigenin-8-*C*-glucoside (vitexin), and luteolin-8-*C*-glucoside (orientin), respectively [46,47,48]. As expected, flavonoid mono-*O*-glycosides displayed a loss of one sugar moiety, whether glucose or rhamnose, with a loss of 162, or 146 Da, respectively. Similarly, this was observed in peaks 40, 50, 53, 62, 70, and 81, showing molecular ions at *m*/*z* 477.1637, 417.1720, 465.1013, 447.1349, 433.1152, and 431.2272 respectively. Subsequently, distinctive fragment ion peaks of aglycone residues were detected at *m*/*z* 315.0915, 255.1957, 303.0407, 301.2084, 271.0582, and 285.0671, respectively, due to a loss of glucose or rhamnose unit. Therefore, they were tentatively identified as isorhamnetin-3-*O*-glucoside [36,42], daidzein-7-*O*-glucoside [49], quercetin-4′-*O*-glucoside [36], quercetin-3-*O*-rhamnoside [50], naringenin-7-*O*-glucoside [44], and Kaempferol-3-*O*-rhamnoside [36,42], respectively.

Peaks 35 and 39: molecular ion peaks [M − H]^−^ were found at *m*/*z* 417.1500 and 433.1359, corresponding to the loss of arabinose and xylose units, respectively, with distinctive fragment ion peaks of the aglycone residues appearing at *m*/*z* 285.0213 and 301.1242 [(M − H)-132]. Hence, they were tentatively identified as kaempferol-3-*O*-arabinoside and quercetin-3-*O*-xyloside, respectively [51]. Quercetin-3-*O*-galactoside (peak 42) had a pseudo molecular ion at *m*/*z* 465.1712 and four characteristic fragment ions at *m*/*z* 303.0500 [(M + H)-galactose], *m*/*z* 285.0687 [(M + H)-galactose-H_2_O], *m*/*z* 247.1283 [(M + H)-galactose-2CO], and *m*/*z* 229.0664 [(M + H)-galactose-H_2_O-2CO] [36]. Gossypetin-8-*O*-glucoside (peak 43) exhibited a molecular ion peak at *m*/*z* 481.1687 with main fragment ions at *m*/*z* 319.1388 [(M + H)-162], confirming the presence of gossypetin aglycone derived from the loss of glucose unit [52]. Compound 44 was identified as baicalein-7-*O*-glucuronide. It exhibited a molecular ion peak at *m*/*z* 445.1336 with a pattern of fragmentation at *m*/*z* 269.1125 [(M − H)-176], corresponding to the loss of glucuronic acid [36,51].

##### Flavonoid Aglycones

Eleven flavonoid aglycones were detected in peaks 41, 46, 66, 69, 74, 75, 76, 79, 80, 83, and 84 with molecular ion peaks at *m*/*z* 305.0639, 319.1014, 287.0536, 285.1326, 269.1132, 303.0495, 315.0496, 269.1017, 289.1795, 317.1166, 301.1427, corresponding to taxifolin, myricetin, luteolin, acacetin, formononetin, quercetin, isorhamnetin, apigenin, eriodictyol, rhamnetin, and kaempferide, respectively. Taxifolin (peak 41) was characterized by a molecular ion peak at *m*/*z* 305.0639 with distinctive fragment ions at *m*/*z* 287.0832 [(M + H)-H2O] [53]. Isorhamnetin (peak 76) displayed a pseudo molecular ion [M − H]^−^ at *m*/*z* 315.0496 with main fragment ions at *m*/*z* 300.0077 and *m*/*z* 301.1315 corresponding to loss of CH_3_ and CH_2_, respectively [36]. Moreover, apigenin (peak 79) demonstrated a molecular ion peak at *m*/*z* 269.1017, with fragment ions at *m*/*z* 254.0987 [(M − H)-CH_3_], *m*/*z* 225.0989 [(M − H)-CO_2_], *m*/*z* 223.0218 [(M − H)-H_2_O-CO], *m*/*z* 151.0379 [(M − H)-C_8_H_6_O], *m*/*z* 117.0294 [(M − H)-C_7_H_4_O_4_], and *m*/*z* 107.0346 [(M − H)-C_8_H_6_O-CO_2_] [36,48].

It is worth mentioning that this is the first report of the metabolites mentioned above in the leaves of *B. armeniaca* using Q-TOF LC/MS/MS, except rutin, afzelin, isoquercitrin, kaempferol, and quercetin, which were the only flavonoids previously reported [8]. *B. armeniaca* was shown to have anti-inflammatory properties, which were attributed to the presence of rutin, isoquercetrin, and afzelin [1]. These compounds were detected by Q-TOF LC/MS/MS analysis in the current study. Thus, the investigation of *B. armeniaca*’s possible anti-Alzheimer activity through inhibition of inflammatory markers was encouraged.

#### 2.2.2. Phenolic Acids

Phenolic acids, which are prevalent in plants, are investigated for their anti-inflammatory, hepatoprotective, antioxidant, antibacterial, cardioprotective, antidiabetic, anticancer, and neuroprotective activities [54]. In *B. armeniaca* extract, six phenolic acids were tentatively detected as caffeic acid, chlorogenic acid, protocatechuic acid, 3-hydroxyanthranilic acid, 4-hydroxybenzoic acid, and 3,4-dihydroxymandelic acid, which were assigned to peaks 12, 15, 16, 25, 29, and 31, respectively. Chlorogenic acid (peak 15) had a molecular ion peak [M − H]^−^ at *m*/*z* 353.0872 with typical fragment ion peaks at *m*/*z* 191.0546 corresponding to [C_7_H_11_O_6_]^−^ residue and *m*/*z* 173.0496 for additional water loss [55]. Caffeic acid (*m*/*z* 179.0549, peak 12), protocatechuic acid (*m*/*z* 153.0263, peak 16), and 4-hydroxybenzoic acid (*m*/*z* 139.0376, peak 29) all showed characteristic fragments at *m*/*z* 135.0395, 109.0180, and 95.0595, respectively, corresponding to neutral loss of CO_2_ (−44 Da) [56]. Interestingly, it is the first report on the detection of phenolic acids in *Bunchosia* genus via Q-TOF LC/MS/MS.

#### 2.2.3. Alkaloids

Intriguingly, three alkaloids were characterized in the studied *B. armeniaca* leaves, designated as trigonelline, harmaline, and caffeine. Trigonelline (*m*/*z* 138.0551, peak 13) produced a fragment ion at *m*/*z* 92.0489 [(M + H)-C_2_H_6_O] derived from an ethyl unit loss (−46 Da) and CO neutral loss (−28 Da) indicated at *m*/*z* 110.0599 [57]. Meanwhile, harmaline (peak 17), with a molecular ion peak at *m*/*z* 215.1401, yielded main fragment ions at *m*/*z* 200.1342 [(M + H)-CH_3_] and *m*/*z* 169.1384 [(M + H)-CO-H_2_O] [58], whereas caffeine (peak 28) displayed a pseudo molecular ion [M + H]^+^ at *m*/*z* 195.1122 with a base peak at *m*/*z* 138.0005 [59].

Previous studies reported the presence of caffeine in *Bunchosia glandulifera* fruit, which is consistent with our findings [32,60]. However, to our knowledge, this is the first report for the detection of trigonelline and harmaline in *Bunchosia*, while trigonelline was previously isolated from the leaves of *Niedenzuella multiglandulosa* (Malpighiaceae) [61].

#### 2.2.4. Polyphenols

As many as nine polyphenolic compounds were presented in *B. armeniaca*, including two stilbenoids (peaks 27 and 78), in addition two flavan-3-ols (peaks 45 and 87), four anthocyanins (peaks 48, 58, 64, and 73), and one chalconoid (peak 49). Cyanidin-3-*O*-rutinoside (peak 48) displayed a pseudo molecular ion [M + H]^+^ at *m*/*z* 595.1667, followed by a peak at *m*/*z* 433.1143 [(M + H)-162] owing to glucose unit loss, and finally a peak at *m*/*z* 287.0569 derived from the cyanidin nucleus (−308). Cyanidin-3-*O*-glucoside (peak 64) showed a molecular ion peak at *m*/*z* 449.1086 and a main fragment ion at *m*/*z* 287.0522 [(M + H)-162] [62]. Astringin (peak 78) was detected by a precursor ion at *m*/*z* 405.1771, giving the characteristic fragment ion at *m*/*z* 243.0687 (−162 Da) [63]. Epicatechin (peak 87) demonstrated a deprotonated ion at *m*/*z* 289.0696 and a distinctive product ion at *m*/*z* 245.0605 due to loss of CO_2_ or CH_2_–CHOH moieties (−44 Da) [56,64]. All polyphenolic compounds in this study were identified for the first time in the genus *Bunchosia* and the plant *B. armeniaca*.

The use of both positive and negative ionization LC-MS provides more comprehensive metabolome coverage than the use of a single mode. Several analytes could only be identified in the negative ion mode, whereas others could only be detected in the positive ion mode. Dual ionization modes were used to identify common differential metabolites such as taxifolin, acacetin, baicalin, isoorientin, astringin, robinin, vitexin, puerarin, tulipanin, spiraeoside, luteolin, narcissin, myricetin, juglalin, daphnetin, chlorogenic acid, acaciin, and hymecromone. The study of the fragmentation pathway of (M − H)^−^/(M + H)^+^ ions in the negative and positive ion modes aided in the identification of these compounds. Most identified constituents were phenolics and flavonoids, which are known to be beneficial to human health. Various studies have documented flavonoids with a wide range of pharmacological activities, including antioxidant, anti-inflammatory, hepatoprotective, and antiangiogenic properties [65], as well as being antidiabetic [66], cardioprotective [67], neuroprotective, and anti-Alzheimer’s disease [68], which are primarily due to their degree of hydroxylation, structural class, other substitutions, conjugations, and degree of polymerization, as well as metal chelation activity [69].

### 2.3. In Vitro Assays

#### 2.3.1. Antioxidant Activity

Antioxidants protect the body from illnesses such as AD, cancer, atherosclerosis, and obesity. The high antioxidant activity of the plant is a prior indication of its potential anti-Alzheimer properties [70]. Plants, vegetables, and fruits are currently the primary sources of antioxidant-active chemical constituents, where their phenolic contents are thought to be responsible for the strength of this antioxidant activity [71]. The antioxidant potential of a plant is defined as the degree to which its antioxidant compound prevents or inhibits the oxidation of biomolecules in the environment [72]. Antioxidant activity is a multi-step process involving multiple mechanisms regulated by various elements that cannot be fully described by a single method. In order to account for the varied mechanisms of antioxidant action, it is necessary to undertake multiple types of antioxidant capacity measurements [73]. Because many plants are complex, it is impossible to assess the antioxidant activity of *B. armeniaca* using one technique. In the current research, three complementary assays were performed (DPPH, ABTS, and FRAP) to evaluate the antioxidant effect of *B. armeniaca* leaves. Fractionation of *B. armeniaca* crude extract resulted in successive fractions with variable degrees of antioxidant activity.

##### DPPH Radical Scavenging Assay

Antioxidant components and radical scavengers are utilized as essential supplemental nutrition that may help protect human health from various diseases [74]. The DPPH radical scavenging test is a helpful approach for assessing the antioxidant properties of natural and synthetic substances. It is among the most potent, efficient, accurate, easy, and reproducible in vitro techniques for determination of this essential activity of single constituents and plant extracts [75]. Using the DPPH test, the radical scavenging activities of the extract and fractions of *B. armeniaca* leaves were evaluated and represented in (Table 2). When compared to BME (IC_50_: 254.3 ± 4.25 μg/mL) and BDMF (IC_50_: 289.0 ± 10.95 μg/mL), BBF demonstrated the highest antioxidant activity (IC_50_: 69.29 ± 1.77 μg/mL) compared to standard Trolox (IC_50_: 24.42 ± 0.87 μg/mL).

According to previous research, the IC_50_ value of *B. armeniaca* fruit’s methanolic extract was 3.443 ± 0.29 mg/mL, while the gallic acid standard had an IC_50_ value of 0.0036 mg/mL [5]. The difference between the current research value and the previous one mentioned above could be due to the plant components tested being different.

##### ABTS Cation Radical Decolorization Assay

The ABTS radical scavenging assay can be used for food and plant extracts, including lipophilic and hydrophilic substances. It depends on the suppressed absorbance of radical cation ABTS+, which has a particular color at 734 nm. As demonstrated in (Table 2), the results of the ABTS assay of the extracts and fractions showed high similarity with the results from the DPPH assay. BBF showed the most potent free ABTS radical-scavenging effect with IC_50_ of 15.59 ± 0.37 μg/mL even compared to standard Trolox (IC_50_: 22.56 ± 0.78 μg/mL). However, BDMF showed relatively moderate results with IC_50_ of 84.91 ± 2.46 μg/mL followed by BME with IC_50_ of 121.27 ± 4.8 μg/mL.

The ABTS assay was used for the first time in this work to investigate the antioxidant properties of *B. armeniaca*. On the other hand, a previous study employed this method to assess *Bunchosia glandulifera*’s antioxidant efficacy, yielding a value of 8928.57 μM of Trolox Equiv./100 g of fruit pulp [60].

##### Ferric Reducing Antioxidant Power (FRAP) Assay

Natural products’ ability to reduce metals is regarded as a key marker of their antioxidant properties [76]. Spectroscopically, the quantity of ferric (Fe^3+^) complex conversion to ferrous (Fe^2+^) form is detected by the color change of the solution based on the compound’s ability for reduction. The increased reduction capability of the studied sample is shown by the higher absorbance of the reaction mixture [73]. FRAP values of *B. armeniaca* extract and its fractions were recorded in (Table 2) and expressed as EC_50_ (µg/mL). BBF had the highest reducing power (100.3 ± 4.26 µg/mL), which was nearly similar to the Trolox standard (96.97 ± 2.73 µg/mL), followed by BDMF (255.4 ± 15.37 µg/mL), while BME was the least efficient (384.7 ± 9.8 µg/mL) to quench iron.

The FRAP assay has never been used to study the antioxidant activity of *B. armeniaca*. However, *Bunchosia glandulifera* was previously proven to have an antioxidant effect, which revealed 19,285.21 FeSO_4_ μM/100 g of fruit pulp [60].

Finally, all antioxidant assays (DPPH, ABTS, and FRAP) revealed similar trends, with BBF having the highest antioxidant capacities. Therefore, these findings confirmed that BBF is enriched with active polar ingredients responsible for the antioxidant properties. The presence of considerable amounts of TPC and TFC in *B. armeniaca* may contribute to its potent antioxidant action [77]. Metabolic profiling of *B. armeniaca* extract revealed an abundance of phenolic compounds, implying that the plant is a powerful antioxidant. The potent radical scavenging potency may be explained by the presence of apigenin, quercetin, luteolin, naringenin [78,79], chlorogenic acid, hyperoside, spiraeoside, quercetin, epicatechin, and procyanidin B2 [80].

#### 2.3.2. Anticholinesterase Activity

AD causes neurodegeneration, which leads to cognitive problems and eventually death. Cholinesterase inhibitors are still used to treat symptoms and cognitive impairment in Alzheimer’s patients [81]. The results of IC_50_ values for acetylcholinesterase (AChE) inhibition of tested samples are represented in (Table 3). Both BBF and BME were potent inhibitors of AChE with IC_50_ values of 16.4 ± 0.81 and 31.5 ± 1.6 μg/mL, respectively, whereas BDMF had no activity when compared to reference donepezil (IC_50_ = 7.89 ± 1.3 μg/mL).

The current in vitro investigation emphasizes, for the first time, the beneficial effect of *B. armeniaca* leaves on AD. It provides a potential indicator for conducting an in vivo study using diverse parameters.

### 2.4. Anti-Alzheimer In Vivo Study

#### 2.4.1. Acute Toxicity

Male and female mice treated with a single dose of 5 g/kg BME showed no signs of toxicity such as mortalities, hair loss, diarrhea, patches of yellow coloration, or behavioral abnormalities. Therefore, the acute lethal toxicity test revealed the safe oral administration of *B. armeniaca* extract.

#### 2.4.2. Effects on Behavioral Activity on Y-Maze

**Figure 3:**
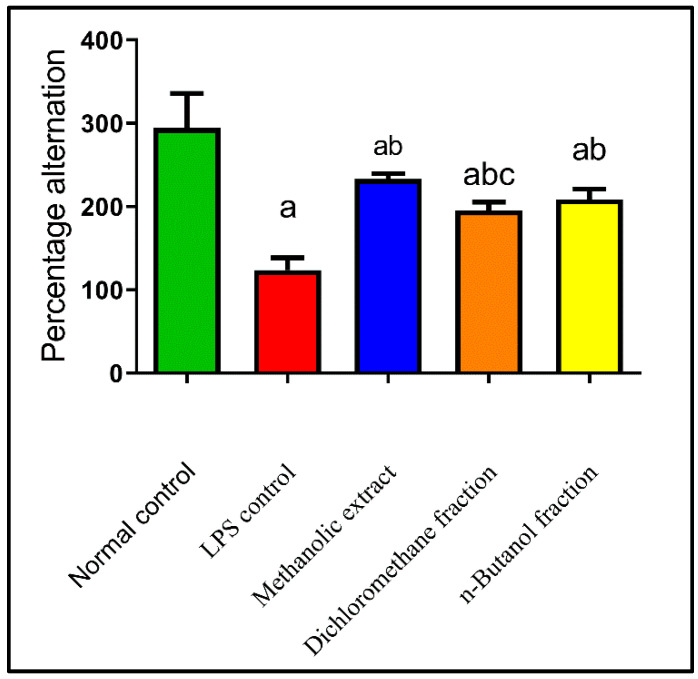
Effects of BME, BDMF, and BBF on behavioral activity on Y-maze in LPS-induced Alzheimer in mice. Data are presented as the mean ± standard error (SE) of *n* = 8 per each group. Statistical analysis was done using one-way analysis of variance followed by Fisher’s LSD test. ^a^ Statistically significant from normal control at *p* < 0.05. ^b^ Statistically significant from LPS control at *p* < 0.05. ^c^ Statistically significant from BME at *p* < 0.05. BME: *B. armeniaca* methanolic extract; BDMF: *B. armeniaca* dichloromethane fraction; BBF: *B. armeniaca* butanol fraction; LPS: lipopolysaccharide.

LPS has been linked to an increase in amyloid beta (Aβ) accumulation, exhibiting pathological abnormalities such as memory deficiency and neuroinflammation. AD is characterized by memory loss, which plays a crucial role in the disease’s progression [82]. LPS caused memory impairment as it decreased Y-maze alteration by 58% as compared to normal control. While BME, BDMF, and BBF improved the behavioral activity on the Y-maze by 88%, 58%, and 68%, respectively, as compared with LPS-administered mice (Figure 3). As a result, BME demonstrated a significant reduction in exploratory behavior followed by BBF, suggesting that they possibly act as learning and memory enhancers.

Numerous previous studies have bolstered the idea that components with a natural origin, primarily flavonoids, have a beneficial role in improving learning and memory functions [83,84,85,86,87]. Furthermore, quercetin has a potent effect on memory and cognitive functions [88,89]. Another previous study demonstrated that quercetin treatment significantly increased the percentage of spontaneous alteration behavior, implying that it enhanced the spatial working memory performance of the LPS-administered mice [90]. Consequently, the flavonoid content in *B. armeniaca* may be responsible for the observed memory and learning improvements.

This is the first report that *B. armeniaca* reduces learning and memory deficiencies in mice. Meanwhile, previous findings showed that *Heteropterys aphrodisiaca*, *Malpighiaceae,* had a similar effect on elderly rats [91].

#### 2.4.3. Effects on NF-κB and IL-1β

In the central nervous system, glial cells maintain the neuron’s normal structure and defend against pathogens [92]. Inflammation induces glial cell activation and releases inflammatory mediators, leading to AD [93]. LPS is a well-known brain macrophage inducer and immune system activator [94]. It stimulates microglial cells, resulting in the production of reactive oxygen species (ROS), nitrogen oxide species (NOS), and a variety of proinflammatory cytokines such as IL-1β, IL-6, tumor necrosis factor (TNF)-α, and interferons (IFNs) [94,95,96]. IL-1β expression plays a significant role in neuroinflammation and is one of the most critical neuropathological variables in neurodegenerative disorders, such as AD. Reduced detrimental effects induced by IL-1β may pave the way for AD treatment [97]. Moreover, NF-κB modulation would provide an approach for AD management by suppressing neuroinflammation and amyloidogenesis [98].

**Figure 4:**
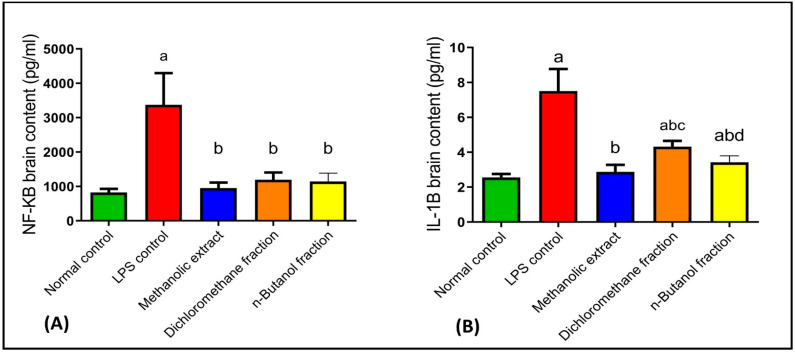
Effects of BME, BDMF, BBF on NF-κB (**A**) and IL-1β (**B**) in LPS-induced Alzheimer in mice. Data are presented as the mean ± standard error (SE) of *n* = 8 per each group. Statistical analysis was done using one-way analysis of variance followed by Fisher’s LSD test. ^a^ Statistically significant from normal control at *p* < 0.05. ^b^ Statistically significant from LPS control at *p* < 0.05. ^c^ Statistically significant from BME at *p* < 0.05. ^d^ Statistically significant from BDMF at *p* < 0.05. BME: *B. armeniaca* methanolic extract; BDMF: *B. armeniaca* dichloromethane fraction; BBF: *B. armeniaca* butanol fraction; LPS: lipopolysaccharide; NF-κB: nuclear factor kappa B; IL-1β: interleukin 1 beta.

LPS injection expressed inflammatory mediators in the brain such as NF-κB and IL-1β by 309% and 193%, respectively, as compared to normal control. Moreover, BME, BDMF, and BBF reduced brain contents of NF-κB by 72%, 65%, and 66% (Figure 4A), and IL-1β by 62%, 42%, and 54% (Figure 4B), respectively, as compared to LPS-administered mice. Interestingly, treatment with BME returned NF-κB and IL-1β in the brain to their normal levels, whereas BBF restored NF-κB level to normal and produced a significant reduction in IL-1β level.

Quercetin reduced the inflammatory cytokines such as TNF-α and NF-κB [99]. Apigenin also significantly attenuated LPS-induced inflammatory response, TNF-α, IL-1β, and IL-6 production as well as suppressed LPS-induced NF-κB stimulation [100]. Moreover, it enhanced expression of brain-derived neurotrophic factor (BDNF) both alone and after an inflammatory stimulus with IL-1β, an action that could be linked to anti-inflammatory and neuroprotective properties [97]. Therefore, the presence of various flavonoids in BME, such as quercetin and apigenin, supports the therapeutic neuroprotective effect of *B. armeniaca* leaves.

The neuroprotective effect of *B. armeniaca,* by suppressing NF-κB and IL-1β, was reported here for the first time. *Hiptage benghalensis*, Malpighiaceae, showed anti-inflammatory effects through inhibition of NF-κB in LPS-stimulated mouse cell line macrophages [101].

#### 2.4.4. Effects on CYP2E1 and GFAP

**Figure 5:**
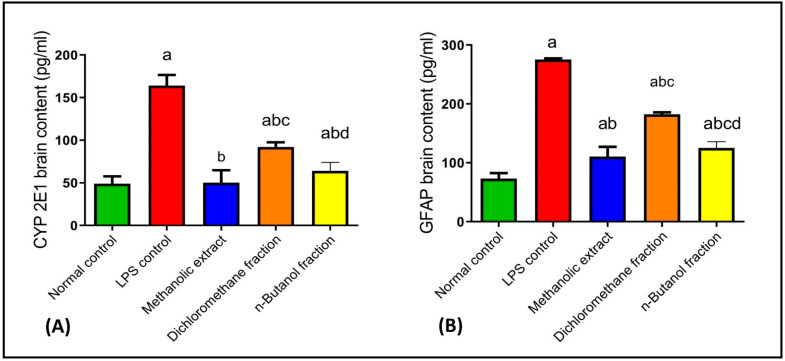
Effects of BME, BDMF, BBF on CYP2E1 (**A**) and GFAP (**B**) in LPS-induced Alzheimer in mice. Data are presented as the mean ± standard error (SE) of *n* = 8 per each group. Statistical analysis was done using one-way analysis of variance followed by Fisher’s LSD test. ^a^ Statistically significant from normal control at *p* < 0.05. ^b^ Statistically significant from LPS control at *p* < 0.05. ^c^ Statistically significant from BME at *p* < 0.05. ^d^ Statistically significant from BDMF at *p* < 0.05. BME: *B. armeniaca* methanolic extract; BDMF: *B. armeniaca* dichloromethane fraction; BBF: *B. armeniaca* butanol fraction; LPS: lipopolysaccharide; CYP2E1: cytochrome P450 2E1; GFAP: glial fibrillary acidic protein.

In previous studies, the activation of glial cells produced ROS, inducing neuronal degeneration [102,103]. CYP2E1 is the most efficient P450 enzyme which initiates NADPH-dependent lipid peroxidation in most biological membranes and generates ROS [104]. Our results revealed that LPS injection elevated oxidative stress as evidenced by the elevation of CYP2E1 brain content by 235% as compared to normal control. While BME, BDMF, and BBF decreased activated CYP2E1 by 69%, 44%, and 61%, respectively, as compared to LPS-administered mice (Figure 5A). Consequently, BME treatment restored CYP2E1 to its normal level, whereas administration of BBF significantly reduced it. Previously, natural flavonoids such as quercetin rescued neuronal degeneration by decreasing activated cytochrome c [90].

Glial cells, including astrocytes and microglia, have been correlated with neurodegenerative disorders such as AD [105]. When astrocytes were activated, they expressed GFAP with amyloid deposition, provoking AD [106]. In the current study, LPS activated astrocytes and elevated GFAP brain expression by 276% as compared to normal control. Whereas BME, BDMF, and BBF decreased GFAP by 60%, 34%, and 54%, respectively, as compared to LPS-administered mice (Figure 5B). Accordingly, BME and BBF were found to be effective in reducing the immunological reactivity of GFAP.

No previous studies reported the effects of any member of the genus *Bunchosia* on CYP2E1 and GFAP.

A previous study found that baicalein had the beneficial effect of diminishing the immunological reactivity of GFAP found in neurons [107]. Resveratrol, a natural polyphenol, has also been identified to have a possible therapeutic effect on lowering GFAP levels, which may help to reduce the risk of neurodegenerative disorders, especially AD [108,109]. As a result, the above-mentioned metabolites in BME might explain the activity of *B. armeniaca* in ameliorating LPS-induced AD.

#### 2.4.5. Histopathological Examination

**Figure 6:**
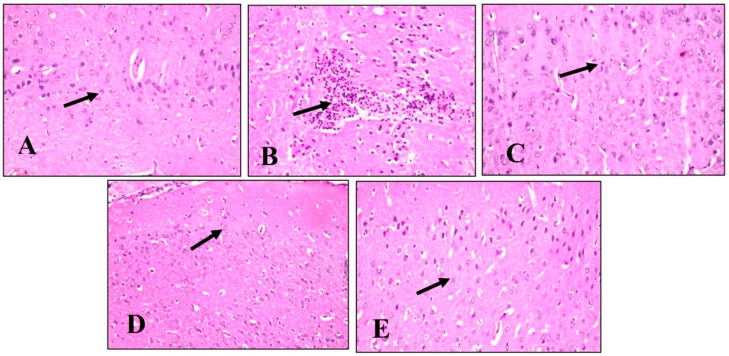
Histopathology of mice brain (Cerebral Cortex sections) with H&E × 40. (**A**) Normal control group showing normal histological findings (Black arrow-normal neuron). (**B**) LPS-administered mice group showing nuclear pyknosis and degeneration of the neurons with focal gliosis (Black arrow). (**C**) BME-administered mice group showing normal histological findings (Black arrow-normal neurons). (**D**) BDMF-administered mice group showing normal histological findings (Black arrow-normal neurons). (**E**) BBF-administered mice group showing normal histological findings (Black arrow-normal neurons). BME: *B. armeniaca* methanolic extract; BDMF: *B. armeniaca* dichloromethane fraction; BBF: *B. armeniaca* butanol fraction; LPS: lipopolysaccharide.

**Figure 7:**
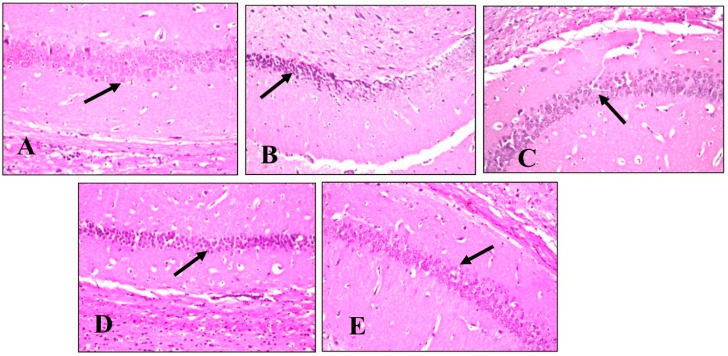
Histopathology of mice brain (Subiculum in hippocampus sections) with H&E × 40. (**A**) Normal control group showing normal histological findings (Black arrow-normal neuron). (**B**) LPS-administered mice group showing nuclear pyknosis and degeneration of some neurons (Black arrow) (**C**) BME-administered mice group showing normal histological findings (Black arrow-normal neuron). (**D**) BDMF-administered mice group showing normal histological findings (Black arrow-normal neuron). (**E**) BBF-administered mice group showing normal histological findings (Black arrow-normal neuron). BME: *B. armeniaca* methanolic extract; BDMF: *B. armeniaca* dichloromethane fraction; BBF: *B. armeniaca* butanol fraction; LPS: lipopolysaccharide.

A histopathological examination of different brain tissues was conducted to provide a supplementary explanation of the brain alterations induced by LPS and the effects of BME, BDMF, and BBF administration. Microscopical examination of the cerebral cortex (Figure 6) revealed no histopathological alteration in normal control sections (Figure 6A). While in mice injected with LPS, cerebral cortex sections had nuclear pyknosis and neuronal degeneration with focal gliosis (Figure 6B). Moreover, the cerebral cortex neurons in mice treated with BME, BDMF, and BBF showed normal histological structure (Figure 6C–E, respectively). Additionally, a histopathological examination of the hippocampus subiculum was carried out, as shown in (Figure 7). The results demonstrated that the hippocampus subiculum of normal control mice and animals treated with BME, BDMF, and BBF had no pathological abnormalities (Figure 7A,C–E, respectively). On the contrary, examined hippocampus subiculum sections from mice administered LPS revealed nuclear pyknosis and degeneration in some neurons (Figure 7B).

**Figure 8:**
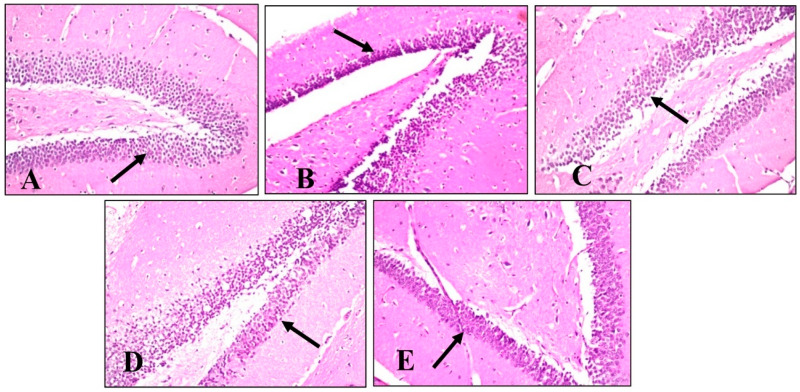
Histopathology of mice brain (Fascia dentata and hilus in hippocampus sections) with H&E × 40. (**A**) Normal control group showing normal histological findings (Black arrow-normal neuron). (**B**) LPS-administered mice group showing nuclear pyknosis and degeneration in most of the neurons (Black arrow). (**C**) BME-administered mice group showing normal histological findings (Black arrow-normal neuron). (**D**) BDMF-administered mice group showing normal histological findings (Black arrow-normal neuron). (**E**) BBF-administered mice group showing normal histological findings (Black arrow-normal neuron). BME: *B. armeniaca* methanolic extract; BDMF: *B. armeniaca* dichloromethane fraction; BBF: *B. armeniaca* butanol fraction; LPS: lipopolysaccharide.

Microscopical examination of fascia dentata and hilus in the hippocampus (Figure 8) displayed no histopathological alteration in normal control sections, and the normal histological structure of the neurons was recorded in (Figure 8A). Most of the hippocampus fascia dentata and hilus neurons of mice administered with LPS revealed nuclear pyknosis with degeneration (Figure 8B). Regarding examined sections of mice treated with BME, BDMF, and BBF, there was no histopathological alteration when compared to the LPS-administered group (Figure 8C–E, respectively).

**Figure 9:**
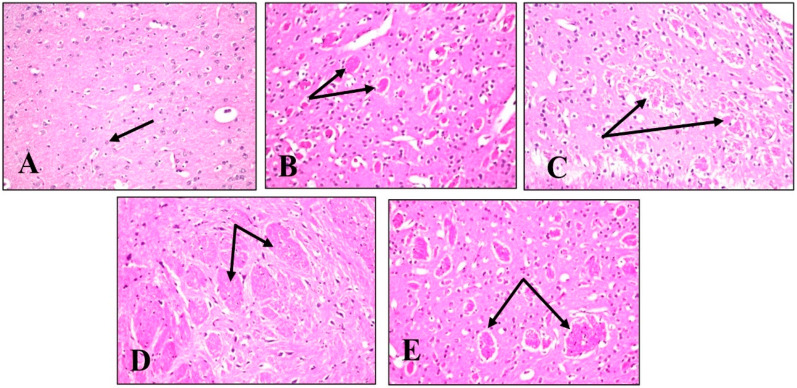
Histopathology of mice brain (Striatum sections) with H&E × 40. (**A**) Normal control group showing normal histological findings (Black arrow-normal neuron). (**B**) LPS-administered mice group showing multiple focal eosinophilic plagues formation (Black arrow). (**C**) BME-administered mice group showing fine eosinophilic plagues formation (Black arrow). (**D**) BDMF-administered mice group showing eosinophilic plagues formation (Black arrow). (**E**) BBF-administered mice group showing focal eosinophilic plagues formation (Black arrow). BME: *B. armeniaca* methanolic extract; BDMF: *B. armeniaca* dichloromethane fraction; BBF: *B. armeniaca* butanol fraction; LPS: lipopolysaccharide.

**Figure 10:**
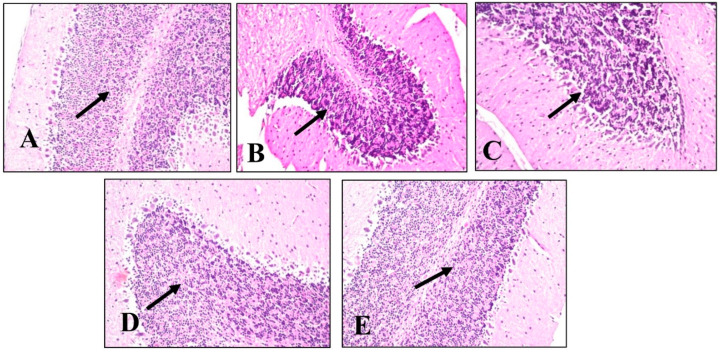
Histopathology of mice brain (Cerebellum sections) with H&E × 40. (**A**) Normal control group showing normal histological findings (Black arrow-normal neuron). (**B**) LPS-administered mice group showing normal histological findings (Black arrow-normal neuron). (**C**) BME-administered mice group showing normal histological findings (Black arrow-normal neuron). (**D**) BDMF-administered mice group showing normal histological findings (Black arrow-normal neuron). (**E**) BBF-administered mice group showing normal histological findings (Black arrow-normal neuron). BME: *B. armeniaca* methanolic extract; BDMF: *B. armeniaca* dichloromethane fraction; BBF: *B. armeniaca* butanol fraction; LPS: lipopolysaccharide.

Similarly, a microscopical investigation of the brain striatum was conducted in (Figure 9). Striatum sections of normal control mice revealed that the histological structure of neurons was normal and there was no histopathological alteration (Figure 9A). On the other hand, sections from LPS-administered mice showed multiple focal eosinophilic plagues formation (Figure 9B). Surprisingly, fine eosinophilic plagues formation was demonstrated in striatum sections of mice treated with BME (Figure 9C), while examined sections of mice treated with BDMF showed the formation of eosinophilic plagues (Figure 9D). Furthermore, striatum sections of mice treated with BBF revealed focal eosinophilic plagues formation (Figure 9E). Ultimately, microscopical examination of the brain’s cerebellum was exhibited in (Figure 10). The normal histological structure of neurons was observed in cerebellum sections of normal control and all other administered mice groups, with no histopathological alteration, as recorded in (Figure 10A–E, respectively).

According to the above histopathological findings, both BME and BBF fractions effectively ameliorate LPS-induced AD disease in mice, which is consistent with biochemical analysis results.

Interestingly, LC-Mass findings in the current study revealed that BME was richer in phenolic constituents. The tentative identification of phenolic compounds such as baicalin, vitexin, caffeic acid, and protocatechuic acid in BME employing positive and negative ion modes may have a significant impact on the management of LPS-induced AD in mice. Baicalin can protect the blood–brain barrier (BBB) from LPS attacks. This action could be mediated by suppressing ROS production as well as the inflammatory response of BBB endothelial cells. So, baicalin has a good protective effect against LPS-induced brain disorders and may be employed as a clinical therapy option for AD [110].

Additionally, vitexin protected transgenic *Caenorhabditis elegans* nematode strains from Aβ proteotoxicity by acting as a neuroprotective agent by extending their life span. As a result, vitexin is a multifactorial agent that has the potential to be a promising therapeutic strategy in AD treatment [111]. Moreover, caffeic acid dramatically improved learning impairments and enhanced cognitive function in AD rats due to inhibition of oxidative stress and inflammation. In addition, compared to the AD model group, caffeic acid treatment caused a significant reduction in acetylcholinesterase efficacy and nitrite formation in the rats with AD [112].

Furthermore, protocatechuic acid (PA) exhibited protective benefits against cognitive impairment in an Aβ-induced AD mouse model. Treatment of higher PA was found to protect against cognitive impairment. Moreover, it had considerably lower lipid peroxidation and NO generation in the brain, kidney, and liver tissues [113].

Finally, the presence of these phenolic metabolites, which have previously been reported as neuroprotective agents, may explain the enhancement therapy of neurodegenerative disorders, particularly AD, observed in *B. armeniaca* leaves. In this regard, the existence of all of these bioactive metabolites may have a synergistic effect on *Bunchosia* extract, which is correlated to its powerful neuroprotective potential. Further bio-guided purification of active fractions of *B. armeniaca* extract is needed in the future to isolate and identify their major active principles.

## 3. Materials and Methods

### 3.1. Plant Material

Fresh *B. armeniaca* leaves have been collected from Mazhar Botanical Garden, Giza, Egypt, during March 2020, and have been generously authenticated by Agriculture Engineer Therease Labib, former Director of El-Orman Botanic Garden in Giza, Egypt, and Plant Taxonomy Consultant at the Agriculture Ministry. A vouchered specimen of *B. armeniaca* with serial number 24.3.2022 II was safely withheld at the Pharmacognosy Department, Faculty of Pharmacy, Cairo University.

### 3.2. Extraction and Fractionation

One and half kilogram of powdered dried *B. armeniaca* leaves were macerated in 90% methanol (7 liters) at ambient temperature. The extraction was repeated three times with addition of fresh solvent. The combined filtered extract was evaporated using a rotary vacuum evaporator at no more than 40 °C, yielding 150 g of dry residue. The crude methanol extract (BME) (140 g) was dissolved with 70 mL of distilled H_2_O. The aqueous solution was successively fractionated by dichloromethane and butanol saturated with water. After evaporation, dichloromethane (BDMF) (40 gm) and butanol (BBF) (45 gm) fractions were obtained. For biological studies and further analysis, the dried extract (10 g) and its fractions were stored at −20 °C.

### 3.3. Estimation of Total Phenolic and Total Flavonoid Contents

Total phenolic content was evaluated by Folin–Ciocalteu colorimetric technique [114]. A calibration curve was prepared using several concentrations of standard methanolic gallic acid solutions (1000, 800, 600, 400, 200, 100, and 50 μg/mL). A volume of 10 µL of extract solution (5 mg/mL methanol) or phenolic standard (1 mg/mL methanol) or blank was added to 100 µL of Folin Ciocalteu reagent and incubated for 5 min. After that, 80 µL of 1 M aqueous sodium carbonate was added and incubated at 37 °C for half an hour. Total phenols were quantified colorimetrically at 630 nm using a microplate reader, FluoStar Omega, in 6 replicates. A calibration curve (y = 0.0031x − 0.0564, R^2^ = 0.9961) was created and data were given in microgram of gallic acid equivalents per milligram of crude extract (µg GA/mg extract).

The aluminum chloride (AlCl_3_) method was developed by [115] to measure the total flavonoid content in the crude extract. For the calibration curve, standard rutin was utilized. One mg of rutin was dissolved in methanol at 1 mg/mL and then diluted to 1000, 600, 400, 200, 100, 50, and 10 μg/mL. The extract was prepared at a concentration of 5 mg/mL in methanol. The extract stock solution (0.5 mL) was added to methanol (1.5 mL), 1% AlCl_3_ (0.1 mL), potassium acetate solution (0.1 mL, 1 M), and distilled H_2_O (2.8 mL). The resultant mixture was kept for incubation at a darkened room temperature for half an hour. The absorbance of the reaction was read at 420 nm by a microplate reader, FluoStar Omega, in 6 replicates. A calibration curve (y = 0.0032x + 0.0398, R^2^ = 0.9981) was drawn and data were given in microgram of rutin equivalents per milligram of crude extract (µg RU/mg extract).

### 3.4. Metabolic Profiling Using Q-TOF LC/MS/MS

#### 3.4.1. Sample preparation

An extract stock solution was made by dissolving 50 mg of freeze-dried methanolic extract in 1 mL of a mixture consisting of distilled water (H_2_O), methanol (MeOH), and acetonitrile (ACN) in a 2:1:1 ratio. The sample was vortexed and ultra-sonicated at 30 kHz for 10 min to obtain complete solubility of the stock solution. Twenty µL of aliquot stock solution were diluted again with 1 mL of H_2_O, MeOH, and ACN in a 2:1:1 ratio, then spun at 10,000 rpm for 5 min, yielding 10 µL (1 µg/mL) for injection. In both positive and negative modes, the sample was injected.

#### 3.4.2. Q-TOF LC/MS/MS Analysis

An ExionLC system (AB Sciex, Framingham, MA, USA) was adjusted with an autosampler system, an in-line filter disc pre-column (0.5 µm × 3.0 mm, Phenomenex, Torrance, CA, USA), and an Xbridge C18 (3.5 µm, 2.1 × 50 mm) column (Waters Corporation, Milford, MA, USA) at 40 °C and a 300 µL/min flow rate for small molecule separation.

For positive mode, solution (A) was composed of 5 mM ammonium formate (NH_4_HCO_2_) in 1% MeOH using formic acid to adjust pH to 3.0, and the mobile phase of solution (B) was comprised of 100% ACN as the mobile phase. Whilst in negative mode, solution (C) was made up of 5 mM NH_4_HCO_2_ in 1% MeOH using sodium hydroxide to adjust pH to 8. The following program was used to do the gradient elution: 10% B for 0–20 min; 90% B for 21–25 min; 10% B for 25.01–28 min; and then 90% B for column equilibration.

#### 3.4.3. Mass Spectrophotometry

The mass spectrometry (MS) was carried out on a Triple TOF 5600+ system with a Duo-Spray source in the ESI mode (AB SCIEX, Concord, ON, Canada). In positive mode, the sprayer capillary and declustering potential voltages were 4500 and 80 eV, respectively, whereas in negative mode, they were −4500 and −80 V. The source temperature was adjusted to 600 °C, the curtain gas at 25 psi, and gases 1 and 2 at 40 psi. The collision energies of 35 V (positive mode) and −35 V (negative mode) were used, along with CE spreading of 20 V and an ion tolerance of 10 ppm. The information-dependent acquisition (IDA) technique was used to operate the TripleTOF5600+. Analyst-TF 1.7.1 was utilized to generate batches for MS and MS/MS data collection. The IDA approach was utilized to simultaneously obtain full-scan MS and MS/MS data. The approach used high-resolution survey spectra ranging from 50 to 1100 *m*/*z*, and the mass spectrometer was programmed to detect a 50-ms survey scan. Later, the top 15 intense ions were chosen for the acquisition of MS/MS fragmentation spectra upon every scan [116].

The MS-DIAL 3.70 open-source software was employed to conduct a non-targeting, small molecule comprehensive sample analysis [117]. ReSpect positive (2737 records) or ReSpect negative (1573 records) databases have been utilized as reference databases, depending on the acquisition technique. The search parameters for the collection of data were adjusted as MS1 and MS2 mass tolerance of 0.01 Da and 0.05 Da, while for detection of a peak, minimum peak height: 100 amplitude; mass slice width: 0.05 Da; smoothing level: 2 scans; minimum peak width: 6 scans; for identification, MS1 and MS2 tolerance: 0.2 Da/each; for alignment, retention time tolerance: 0.05 min, and MS1 tolerance of 0.25 Da.

### 3.5. In Vitro Antioxidant Assays

#### 3.5.1. DPPH Radical Scavenging Assay

The free radical potential of the extract, fractions, and standards (BME, BDMF, BBF, and Trolox) was determined spectrophotometrically utilizing the DPPH procedure described by [118]. In brief, 100 μL of fresh DPPH solution (0.1% in MeOH) and 100 μL of the dissolved methanolic sample were pipetted into 96 wells plate (*n* = 6). The reaction was gently mixed and kept for 30 min of incubation in the dark at ambient temperature. The resultant intensity reduction in DPPH color was determined at 540 nm before and after the reaction using a microplate reader, FluoStar Omega, at the termination of the incubation period, and data are represented as mean ± standard deviation (SD). The IC_50_ values for the radical scavenging activities of the extract and fractions are expressed as 50% inhibition concentrations (μg/mL) of DPPH radicals in the reaction.

#### 3.5.2. ABTS Cation Radical Decolorization Assay

The ABTS radical cation was determined spectrophotometrically using the procedure of [119] with slight changes to be performed in microplates. An ABTS solution was prepared by dissolving 192 mg of ABTS in distilled H_2_O and completing the volume to 50 mL with distilled H_2_O. One mL of the prepared ABTS solution was added to potassium persulfate (17 μL, 140 mM) and kept in the dark for one day. The final ABTS dilution was obtained by diluting the reaction mixture (1 mL) to MeOH (50 mL) to achieve an absorbance of 0.700 ± 0.030 at 734 nm. In a 96-microplate (*n* = 6), 190 μL of fresh ABTS solution was added to 10 μL of each sample (BME, BDMF, BBF), and the plate was kept for 2 h at a dark ambient temperature for incubation. At the termination of the incubation period, the intensity reduction in ABTS color was recorded at 734 nm before and after the reaction using a microplate reader, FluoStar Omega, and data are represented as mean ± SD. The IC_50_ values for the radical scavenging activities of the extract and fractions are expressed as 50% inhibition concentrations (μg/mL) of the ABTS radicals in the reaction.

#### 3.5.3. Ferric Reducing Antioxidant Power Assay

The FRAP technique was carried out in microplates with slight modifications as described by [120,121]. Briefly, a fresh FRAP solution was prepared shortly before the reaction in a 10:1:1 *v*/*v*/*v* ratio, consisting of acetate buffer (300 mM, PH = 3.6), tripyridyltriazine (TPTZ, 10 mM) in HCl (40 mM), and FeCl_3_ (20 mM), respectively. In a 96-microplate (*n* = 6), 190 μL of fresh FRAP solution was added to 10 μL of each sample (BME, BDMF, BBF), and the plate was kept for 30 min of incubation at a dark ambient temperature. The resulting blue color was observed at 593 nm at the termination of the incubation period. The FRAP test measured the absorbance change generated by electron-donating antioxidants converting a colorless oxidized Fe^+3^-TPTZ complex to a blue-colored Fe^+2^ molecule in acidic conditions. Data are represented as mean ± SD.

### 3.6. In Vitro Acetylcholinesterase Inhibition Activity

The inhibition of AChE activity was evaluated according to [122], which was modified by [123]. For the activity measurement, AChE was utilized as the enzyme, with acetylthiocholine iodide (AChI) as the substrate, 5,5-Dithiobis-(2-nitrobenzoic acid) (DTNB) as a coloring agent, and donepezil as a positive control. Briefly, sodium phosphate buffer (150 μL, 100 mM, PH = 8.0) was mixed with 10 μL of each sample (BME, BDMF, BBF) dissolved in methanol with a range of concentrations (1000 to 7.81 μg/mL). The AChE solution (20 μL, 5.32 × 10^−3^ U) was then mixed and kept for 15 min of incubation at ambient temperature. Subsequently, DTNB (10 μL, 0.5 mM) and AChI (10 μL, 0.71 mM) were mixed for the initiation of the reaction. The hydrolysis of AChE can be evaluated by the determination of the colorful product 5-thio-2-nitrobenzoate anion formed by DTNB reaction with thiocholine, which is liberated by AChI hydrolysis. The absorbance of colored product was read at 412 nm using a Multiplate Reader.

### 3.7. In Vivo Study

#### 3.7.1. Acute Toxicity Study

For this investigation, male and female Swiss mice weighing 20–30 gm were procured from the animal house laboratory at the National Research Centre (NRC), Cairo, Egypt. Mice were kept in a hygienic laboratory environment for seven days before the start of the biological experiment (adaptation period), held in a well-ventilated box at 22 ± 20 °C for a 12-h lighting and darkness cycle. A natural baseline diet was given to the mice. Diets and water were provided *ad libitum*, with water readily accessible. They were managed in compliance with animal experiment guidelines approved by the Ethical Committee of Medical Research, NRC, Cairo, Egypt.

Acute toxicity was carried out in compliance with the guidelines of the World Health Organization (WHO) for assessing the safety and efficacy of herbal remedies. Four groups of forty Swiss mice were created. Groups one and two: control male and female mice (ten for each group) were orally administered with saline. Groups three and four: male and female mice (ten for each group) were orally administered with a single dose of BME in graded doses up to 5 g/kg. The animals were observed daily for signs of behavioral changes for two weeks [124].

#### 3.7.2. Evaluation of Anti-Alzheimer Activity

##### Animals

Fifty male Swiss mice weighing 20–35 gm were chosen for the investigation. They were held in filter-top plastic cages under hygienic conditions, with 12-h light and 12-hour dark cycles and 50% humidity at 28 °C. Unnecessary disturbance of animals was avoided. Squeezing, pressure, and abrasive maneuvers were avoided when dealing with the animals. Throughout the experiment, mice were fed a standard pellet meal, and water was allowed *ad libitum*. The laboratory animal experiments were conducted to comply with the recommendations of the Guide for the Care and Use of Laboratory Animals of the National Research Centre, Egypt, and the National Institutes of Health (NIH No. 85:23 revised 1985) in accordance with the guidelines provided by the CPCSEA and the World Medical Association Declaration of Helsinki on Ethical Principles for Studies Involving Experimental Animals. This study was approved by the Ethics Committee of the Faculty of Pharmacy, Cairo University, Egypt [Protocol approval number: MP (3139)].

##### Chemicals and Kits

Lipopolysaccharide (LPS) was purchased from Sigma Chemical. Nuclear factor kappa B (NF-κB), interleukin 1 beta (IL-1β), cytochrome P450 2E1 (CYP2E1), and glial fibrillary acidic protein (GFAP) were obtained from (SunLong Biotec Co., Ltd., Hangzhou, China) using specific ELISA kits.

##### Experimental Design

Mice were randomly allocated to five groups (ten each): normal control group; positive control group (LPS 250 µg/kg/once daily for 7 days; intraperitoneal (IP)) [125]; BME-treated group; BDMF-treated group; and BBF-treated group (each 200 mg/kg/day; orally by gastric tube for 7 days) [1].

##### Effects on Y-Maze in LPS-Induced Alzheimer in Mice

Y maze spontaneous alternation test was performed 24 h after the last treatment dose, according to [126]. An animal must remember which arm it has previously entered to be able to alternate its choice on a subsequent trial. Behavioral pharmacologists and others have praised spontaneous alternation behavior as a fast and comparatively basic memory test in recent years [127]. The experiment was conducted in a three-armed Y maze-shaped apparatus. A, B, or C were labeled on each arm. The test is divided into two parts, the first of which is the training phase. The mouse was permitted free movement throughout the maze for eight minutes. One day later, the mouse was allowed eight minutes of movements while its motion was recorded. The letter of the mouse was recorded down every time it entered an arm with all of its limbs inside. The alternations number refers to the successive entries into three separate arms in overlapping triplet sets (for example, ABCBACA = 3). Total arm entries are merely the total number of arms entered (for example, ABCBACA= 7). The alternation percentage was determined using the formula below [128]:

Number of alternations/(Total arm entries −2) ∗ 100

##### Tissue Biochemical Analysis

Firstly, mice were anesthetized using an IP injection of ketamine at a dose of 80–100 mg/kg. After they achieved a deep anesthetic state, mice were sacrificed by decapitation. Each mouse’s brain was promptly dissected and washed with phosphate-buffered saline (PBS) to eliminate extra blood. Weighed pieces were homogenized in PBS with the MPW-120 homogenizer, Med Instruments, Poland, to obtain a 20% homogenate, then they were kept overnight at −20 °C. Using a refrigerated centrifuge (laborzentrifugen, 2K15, Sigma, Osterode am Harz, Germany), the homogenates were spun for five minutes at 5000× *g* [129]. The supernatant was removed immediately and assayed for brain contents of NF-κB, IL-1β, CYP2E1, and GFAP. They were determined using an ELISA kit (SunLong Biotec Co., Ltd., Hangzhou, China).

Standards and samples were added to wells containing immobilized antibodies specific for rat NF-κB, IL-1β, CYP2E1, and GFAP and kept for 30 min of incubation at 37 °C. After washing, horseradish peroxidase-conjugated streptavidin was added to the wells, kept for 30 min of incubation at 37 °C, and then washed for a second time. The wells were filled with tetramethylbenzidine (TMB) substrate solution and kept for 15 min of incubation at 37 °C; a color was produced in proportion to the quantity of NF-κB, IL-1β, CYP2E1, and GFAP bound. A stop solution was added to the wells to terminate the color development, and color intensity was detected at 450 nm after 10 min [130].

##### Histopathological Examination

Autopsy specimens were obtained from mice brains, divided into several groups and preserved in 10% formol saline for a day. Subsequently, rinsing with tap water, dehydration was achieved by alcohol serial dilutions (methyl, ethyl, and absolute ethyl alcohol). Samples were cleaned with xylene and immersed in paraffin for a day at 56 °C in a hot air oven. Paraffin beeswax tissue blocks were made using a sledge microtome and sectioned at a thickness of 4 μM. The tissue sections were mounted on glass slides, deparaffinized, and stained with hematoxylin and eosin (H&E) for routine inspection under a light electric microscope [131].

### 3.8. Statistical Analysis

DPPH and ABTS assay data were estimated by Microsoft Excel^®^. IC_50_ value was computed by GraphPad Prism 5^®^ through converting the concentrations to their logarithmic value and choosing a nonlinear inhibitor regression equation (log (inhibitor) vs. normalized response—variable slope equation) [132]. FRAP assay data were determined by Microsoft Excel^®^. EC_50_ value was computed by GraphPad Prism 5^®^ using plotting log concentration values of the compound/extract against the normalized absorbances (as % maximum activity, where the greatest reading was normalized to be 100%). The EC_50_ was calculated using a non-linear response regression equation (log (concentration) vs. normalized response—variable slope equation). AChE inhibition activity was performed in triplicate. Data were recorded as mean ± SD, and the results were expressed as an IC_50_ value (μg/mL). Statistical analysis was conducted using GraphPad Prism (Ver. 5.0). In vivo study data are expressed as mean ± standard error (SE). One-way analysis of variance (ANOVA) was utilized for comparing various groups, followed by Fisher’s LSD test for multiple comparisons. These statistical tests were carried out using GraphPad Prism software, version 5 (Inc., California, USA). The difference was considered significant when *p* < 0.05.

## 4. Conclusions

The current results highlight the possible use of *Bunchosia armeniaca* plant as a promising candidate for ameliorating AD. Methanolic extract of *B. armeniaca* leaves exerts a significant neuroprotective effect. Among its fractions, the butanol fraction demonstrated profound antioxidant and anti-Alzheimer properties, both in vitro, through inhibition of acetylcholinesterase, and in vivo, by improving behavioral alterations and enhancing several biochemical parameters. These activities are most likely due to the high content of polyphenols and flavonoids, as evidenced by metabolic profiling results. This work encourages further investigation of *B. armeniaca* leaves to explore its promising neuroprotective potential, to isolate its antioxidant components, and to assess their in vivo antioxidant activity in the future.

## Figures and Tables

**Figure 1:**
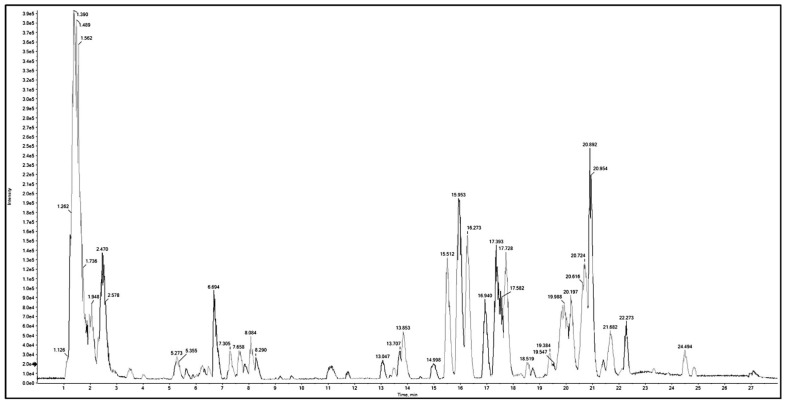
Base peak chromatogram (BPC) of Q-TOF LC/MS/MS analysis of *B. armeniaca* methanolic extract (BME) in positive mode.

**Figure 2:**
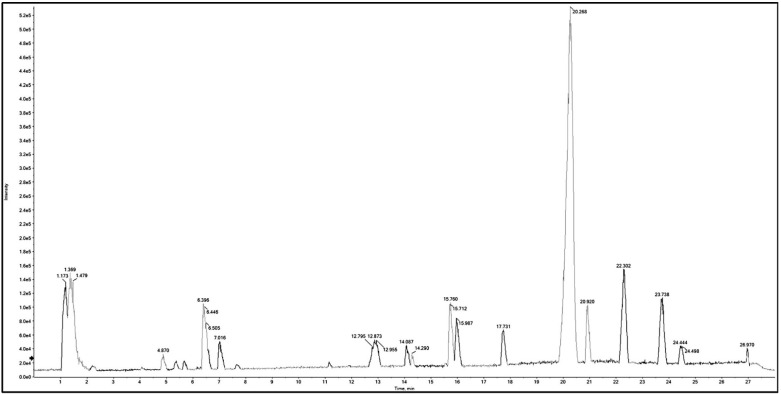
Base peak chromatogram (BPC) of Q-TOF LC/MS/MS analysis of *B. armeniaca* methanolic extract (BME) in negative mode.

**Table 1:** Peak assignments of metabolites in *B. armeniaca* methanolic extract (BME) by Q-TOF LC/MS/MS.

No.	RT (min)	Mol. Ion *m*/*z*		Identified Compound	Molecular Formula	Error (ppm)	Fragment Ions
		[M + H]^+^ [M]^+^	[M − H]^−^ [M]^−^				
Amino acids							
1	0.98	163.1108		5-Hydroxylysine	C_6_H_14_N_2_O_3_	2.8	145.1001, 128.0689, 117.0568, 100.0812
2	1.16	156.0502		L-Histidine	C_6_H_9_N_3_O_2_	0.7	110.0036, 93.0434, 83.0586, 68.9831, 66.0199
6	1.21		102.0549	3-Aminoisobutyric acid	C_4_H_9_NO_2_	0.6	Not fragmented
7	1.22		146.0448	L-Glutamic acid	C_5_H_9_NO_4_	0.8	128.0347, 102.0588, 100.0358, 91.0552, 72.0092
9	1.27	104.1065		2-Aminoisobutyric acid	C_4_H_9_NO_2_	1	87.0409, 60.0787, 58.0643, 56.0483
11	1.34	205.0677	203.0818	L-Tryptophan	C_11_H_12_N_2_O_2_	3	188.0687, 170.0324, 159.0884, 144.0814, 142.0646, 132.0801, 130.0656, 118.0644, 74.0168
14	1.41	147.0665		L-Glutamine	C_5_H_10_N_2_O_3_	−1.2	130.0494, 129.0208, 84.0424, 56.0485, 55.0151
18	1.52		130.0860	L-Hydroxyproline	C_5_H_9_NO_3_	6.6	113.04095
19	1.56	182.0796		Tyrosine	C_9_H_11_NO_3_	4.7	147.0424, 136.0729, 123.0443, 119.0515, 95.0484, 91.0518, 77.03807
20	1.76	130.0493		L-Pyroglutamic acid	C_5_H_7_NO_3_	1	112.1001, 84.08121, 70.06413, 56.05059
21	1.85	132.1025		D-Allo-isoleucine	C_6_H_13_NO_2_	−4.8	86.0952, 69.0702, 57.0554
22	2.15	166.0860		Phenylalanine	C_9_H_11_NO_2_	0.4	131.0498, 120.0799, 103.0523, 91.0546, 77.0381
23	2.16	182.0818		L-Methionine sulfone	C_5_H_11_NO_4_S	−1.4	136.0399, 56.0465
Alkaloids							
13	1.39	138.0551		Trigonelline	C_7_H_7_NO_2_	−0.6	110.0599, 94.0651, 92.0489, 78.8031
17	1.51	215.1401		Harmaline	C_13_H_14_N_2_O	−2.5	200.1342, 169.1384, 156.0641, 70.0640
28	3.37	195.1122		Caffeine	C_8_H_10_N_4_O_2_	3.3	163.0349, 138.0005, 95.0816, 70.0642
Phenolic acids							
12	1.35		179.0549	Caffeic acid	C_9_H_8_O_4_	3.5	161.0414, 135.0395, 133.0644, 117.0399, 109.0569
15	1.43	355.1033	353.0872	Chlorogenic acid	C_16_H_18_O_9_	1.1	284.0475, 191.0546, 179.0548, 173.0496
16	1.45		153.0263	Protocatechuic acid	C_7_H_6_O_4_	−3.9	135.0166, 112.9867, 109.0180, 84.9922, 78.95688
25	2.33	154.0476		3-Hydroxyanthranilic acid	C_7_H_7_NO_3_	10.9	136.0369, 108.0399, 81.0658, 80.0468, 53.0369
29	3.40	139.0376	137.0236	4-Hydroxybenzoic acid	C_7_H_6_O_3_	3.1	121.0227, 111.0877, 95.0595, 67.0373
31	3.97		183.1393	3,4-Dihydroxymandelic acid	C_8_H_8_O_5_	−0.4	Not fragmented
Flavonoid triglycosides							
47	6.26	741.2262	739.2115	Robinin (Kaempferol-3-*O*-robinoside-7-*O*-rhamnoside)	C_33_H_40_O_19_	−0.6	723.23969, 595.1675, 449.10574, 433.1141, 287.0566, 147.0656, 129.0527, 71. 0485
Flavonoid diglycosides							
32	4.02	595.1846		Saponarin (Apigenin-6-*C*-glucoside -7-*O*-glucoside)	C_27_H_30_O_15_	−7.4	Not fragmented
34	4.74		593.1525	Kaempferol-7-*O*-neohesperidoside	C_27_H_30_O_15_	−1.5	503.12836, 473.0619, 431.09586, 285.0508
38	5.20	593.2739	591.2206	Acaciin (Acacetin 7-*O*-rutinoside)	C_28_H_32_O_14_	1.5	575.5092, 503.2012, 473.22516, 447.2165, 431.18842, 285.1368, 267.12045
52	6.66	609.1923		Diosmin (Diosmetin 7-*O*-rutinoside)	C_28_H_32_O_15_	0.4	591.2541, 549.2500, 463.0887, 447.2338, 331.2256, 301.2999, 184.0679
57	7.03		609.1534	Luteolin-3′, 7-di-*O*-glucoside	C_27_H_30_O_16_	−0.6	563.2207, 471.0513, 447.2244, 430.9771, 285.0479, 267.0310, 112.9840
59	7.23		595.2007	Neoeriocitrin (Eriodictyol-7-*O*-neohesperidoside)	C_27_H_32_O_15_	1.1	577.1651, 449.1341, 287.1189
61	7.32	595.1644		Poncirin (Isosakuranetin-7-*O*-neohesperidoside)	C_28_H_34_O_14_	1.6	449.1119, 433.1142, 431.1005, 287.0566, 147.0629, 85.0291
65	7.42	625.1824	623.1544	Narcissin (Isorhamnetin-3-*O*-rutinoside)	C_28_H_32_O_16_	−4.2	607.0872, 505.21542, 479.1170, 463.1479, 317.0589, 147.0355, 85.0486
Flavonoid monoglycosides							
35	4.85	419.1354	417.1500	Juglalin (Kaempferol-3-*O*-arabinoside)	C_20_H_18_O_10_	8.6	387.1057, 354.9223, 343.2181, 285.0213
39	5.22		433.1359	Reynoutrin (Quercetin-3-*O*-xyloside)	C_20_H_18_O_11_	0.1	301.1242, 283.0096
40	5.24		477.1637	Isorhamnetin-3-*O*-glucoside	C_22_H_22_O_12_	−5.8	431.1520,429.1815,401.1725, 315.0915, 285.0234, 227.0368
42	5.70	465.1712		Hyperoside (Quercetin-3-*O*-galactoside)	C_21_H_20_O_12_	0	345.0162, 375.0731, 303.0500, 285.0687, 247.1283, 229.0664, 153.0509
43	5.87	481.1687		Gossypin (Gossypetin-8-*O*-glucoside)	C_21_H_20_O_13_	−0.1	391.0742, 361.1997, 319.1388, 169.1997
44	6.02	447.1446	445.1336	Baicalin (Baicalein-7-*O*-glucuronide)	C_21_H_18_O_11_	0.9	427.1506, 325.1046, 293.0912, 269.1125, 175.0739, 161.0405, 149.0441, 113.1216, 101.0233
50	6.50	417.1720		Daidzin (Daidzein-7-*O*-glucoside)	C_21_H_20_O_9_	2.1	199.2364, 255.1957
51	6.56	449.1086	447.0901	Isoorientin (Luteolin-6-*C*-glucoside)	C_21_H_20_O_11_	−3.3	431.0888, 359.0845, 329.0694, 287.0613, 251.1983
53	6.69	465.1013	463.0841	Spiraeoside (Quercetin-4′-*O*-glucoside)	C_21_H_20_O_12_	1.1	432.0887, 303.0407, 285.1937, 229.0478, 163.0349, 153.0134, 137.0204
54	6.83	433.1491	431.0963	Vitexin (Apigenin-8-*C*-glucoside)	C_21_H_20_O_10_	0	415.1018, 397.0891, 343.2051, 337.0742, 313.0685, 297.0719, 283.0607, 271.0547, 121.0298
56	7.02		593.1286	Tiliroside	C_30_H_26_O_13_	0.4	307.0884, 285.0191, 284.0302, 255.0293, 227.0339, 151.0142
60	7.28	417.1721	415.1568	Puerarin (Daidzein-8-*C*-glucoside)	C_21_H_20_O_9_	0.9	397.1623, 369.1387, 295.0405, 267.0535, 253.1208, 179.0535
62	7.33		447.1349	Quercitrin (Quercetin-3-*O*-rhamnoside)	C_21_H_20_O_11_	−1.4	429.2153, 401.1227, 301.2084, 271.0723, 242.9609, 163.0403, 151.0251
63	7.35	449.1419		Orientin (Luteolin-8-*C*-glucoside)	C_21_H_20_O_11_	−0.9	359.1362, 329.0694, 287.1072
70	7.90		433.1152	Prunin (Naringenin-7-*O*-glucoside)	C_21_H_22_O_10_	−5.4	415.2054, 313.011, 271.0582, 256.9158, 228.9442
81	15.98		431.2272	Afzelin (Kaempferol-3-*O*-rhamnoside)	C_21_H_20_O_10_	0.9	340.9432, 285.0671, 255.9679, 163.04681 151.9852, 133.0201
Flavonoid aglycone							
41	5.62	305.0639	303.0313	Taxifolin	C_15_H_12_O_7_	0.9	287.0832, 149.0264, 127.0152
46	6.17	319.1014	317.0557	Myricetin	C_15_H_10_O_8_	−0.4	289.1131, 245.0399, 181.10976,153.0727, 139.04166, 111.04249
66	7.59	287.0536	285.1381	Luteolin	C_15_H_10_O_6_	2.7	269.16568, 259.05075, 241.13669, 219.0949, 231.06117, 153.0167, 135.0855
69	7.74	285.1326	283.1028	Acacetin (Linarigenin)	C_16_H_12_O_5_	−1.3	270.0095, 267.1167, 255.0842, 242.0842, 213.0459
74	9.02	269.1132		Formononetin	C_16_H_12_O_4_	5	254.15146, 206.1075
75	9.74	303.0495		Quercetin	C_15_H_10_O_7_	−0.7	285.0459, 275.0452, 259.1176, 257.0400, 247.0503, 229.0478, 195.0265, 165.1292, 153.0968
76	11.40		315.0496	Isorhamnetin	C_16_H_12_O_7_	2.6	301.1315, 300.0077, 283.0141, 151.0951
79	14.29		269.1017	Apigenin	C_15_H_10_O_5_	−0.3	254.0987, 225.0989, 223.0218, 151.0379, 117.0294, 107.0346
80	15.11	289.1795		Eriodictyol	C_15_H_12_O_6_	0.6	201.88115, 179.1848, 153.18488, 135.0888
83	19.94	317.1166		Rhamnetin	C_16_H_12_O_7_	−2.5	302.1045, 289.0751, 149.0220, 121.0259
84	20.20	301.1427		Kaempferide	C_16_H_12_O_6_	−2	286.1130, 272.1159, 201.0075, 153.980, 135.0079
Coumarin							
30	3.45	177.0744	174.9555	Hymecromone	C_10_H_8_O_3_	4.3	121.0974, 149.0219, 105.0720, 103.0552, 93.0764, 91.0520, 77.0383
33	4.05	193.0855		Scopoletin	C_10_H_8_O_4_	0.9	133.0619, 115.0577
36	4.87		339.2013	Aesculin (Esculin)	C_15_H_16_O_9_	−0.9	295.1424, 271.0700, 203.0817, 177.0179, 159.0881, 133.0132, 119.0471
72	8.10	179.1077	177.0555	Daphnetin (7,8-dihydroxycoumarin)	C_9_H_6_O_4_	−0.2	133.1012, 107.0848, 105.0719, 77.0357
Polyphenol							
27	2.76	229.1544		Resveratrol	C_14_H_12_O_3_	0.6	214.0611, 187.0538, 185.0874, 170.0767, 145.0812
45	6.05	579.1827		Procyanidin B2	C_30_H_26_O_12_	0.9	Not fragmented
48	6.36	595.1667		Antirrhinin (cyanidin-3-O-rutinoside)	C_27_H_31_O_15_	−0.4	577.2179, 449.1097, 433.1143, 287.0569, 271.5852, 163.0382, 85.0287
49	6.44		449.1088	Marein (Okanin-4′-O-glucoside)	C_21_H_22_O_11_	−0.8	431.1997, 401.1803, 287.0105, 153.0238, 135.0119
58	7.15	611.1625	609.1437	Tulipanin (Delphinidin 3-O-rutinoside)	C_27_H_31_O_16_	−1.5	465.1013, 449.1120, 303.0505, 285.0316, 257.0490, 243.0799, 229.0521, 173.0621, 165.0679, 145.0493, 129.0269
64	7.38	449.1086		Chrysanthemin (Cyanidin-3-O-glucoside)	C_21_H_21_O_11_	−0.4	287.0522, 259.0772, 206.0878, 149.0908, 143.0493
73	8.14	493.1316		Primulin (Malvidin-3-galactoside)	C_23_H_25_O_12_	3.3	373.2349, 331.0705, 315.0639
78	12.79	407.1350	405.1771	Astringin	C_20_H_22_O_9_	−1.1	243.0687, 225.0550, 201.0578, 179.0568, 161.0431, 159.0354
87	20.92		289.0696	Epicatechin	C_15_H_14_O_6_	−0.3	245.0605, 205.0152, 179.0347, 108.9038
Miscellaneous compounds							
3	1.17		168.0293	Pyridoxine	C_8_H_11_NO_3_	−0.4	Not fragmented
4	1.18		133.0128	Malic acid	C_4_H_6_O_5_	2	115.0044, 89.0230, 87.0105, 72.9940, 71.0132, 59.0171
5	1.20		191.0570	Citric acid	C_6_H_8_O_7_	−2.2	173.0195, 130.9981, 129.0160, 111.0064, 87.0103, 85.0325
8	1.23		135.0288	γ-Terpinene	C_10_H_16_	−0.5	117.0078
10	1.32		122.0241	Niacin (Nicotinic acid)	C_6_H_5_NO_2_	0.4	78.0322, 61.9938
24	2.30		225.0666	Carnosine	C_9_H_14_N_4_O_3_	−7.4	130.0676, 113.0907, 89.0232
26	2.60	220.1170	218.1039	Pantothenate (Pantothenic acid)	C_9_H_17_NO_5_	1.5	202.1061, 184.0965, 124.0741, 116.0294, 98.0232, 90.0575, 85.0657
37	5.07	153.0536		*O*-Anisic acid	C_8_H_8_O_3_	3.2	105.0371, 92.0273, 79.0541, 77.0380, 51.0239
55	7.00	377.1448		Riboflavin	C_17_H_20_N_4_O_6_	1.3	243.0886, 198.0633, 172.0824
67	7.61	227.1278		Hedione (Methyl dihydrojasmonate)	C_13_H_22_O_3_	0.9	209.1732, 195.0892, 167.1095, 153.1283
68	7.66	225.1952		Methyl Jasmonate	C_13_H_20_O_3_	3.3	194.9544, 165.0533, 143.1167, 55.0549
71	8.04	411.1765		γ-Tocotrienol	C_28_H_42_O_2_	−0.5	409.8878, 242.1209
77	11.85	169.1223		Pyridoxamine	C_8_H_12_N_2_O_2_	0.1	Not fragmented
82	19.90	149.0222		Cinnamic acid	C_9_H_8_O_2_	4.4	131.0469, 105.0635, 103.0468, 75.0215, 53.0020
85	20.26		277.2150	γ-Linolenic acid	C_18_H_30_O_2_	6.7	259.2028, 233.2145, 231.2238, 205.1942, 59.0166
86	20.80	281.1759		Linoleic acid	C_18_H_32_O_2_	−3.1	263.1773, 221.1796, 123.0819, 83.0433
88	22.58	241.1969		Anserine	C_10_H_16_N_4_O_3_	−4.5	166.0745, 163.1030, 112.0812

**Table 2:** Antioxidant activity of BME, BDMF, and BBF by DPPH, ABTS, and FRAP assays.

Extract/Fractions	DPPH Assay IC_50_ µg/mL	ABTS Assay IC_50_ µg/mL	FRAP Assay EC_50_ µg/mL
BME	254.3 ± 4.25	121.27 ± 4.8	384.7 ± 9.8
BDMF	289.0 ± 10.95	84.91 ± 2.46	255.4 ± 15.37
BBF	69.29 ± 1.77	15.59 ± 0.37	100.3 ± 4.26
Trolox	24.42 ± 0.87	22.56 ± 0.78	96.97 ± 2.73

Data are expressed as mean ± standard deviation (SD). BME: *B. armeniaca* methanolic extract; BDMF: *B. armeniaca* dichloromethane fraction; BBF: *B. armeniaca* butanol fraction; DPPH: 2,2-diphenyl-1-picryl-hydrazyl-hydrate; ABTS: 2,2-azino bis (3-ethylbenzothiazoline-6-sulphonic acid; FRAP: ferric reducing power.

**Table 3:** The acetylcholinesterase (AChE) enzyme inhibition activity of BME, BDMF, and BBF.

Extract/Fractions	AChE (IC_50_ µg/mL)
BME	31.5 ± 1.6
BDMF	>1000
BBF	16.4 ± 0.81
Donepezil	7.89 ± 1.3

Data are expressed as mean ± standard deviation (SD). BME: *B. armeniaca* methanolic extract; BDMF: *B. armeniaca* dichloromethane fraction; BBF: *B. armeniaca* butanol fraction.

## Data Availability

The data presented in this study are available on request from the first or corresponding authors.

## Supplementary Materials

The following supporting information can be downloaded at: https://www.mdpi.com/article/10.3390/plants11141792/s1, Figure S1: Base peak chromatogram (BPC) of Q-TOF LC/MS/MS analysis of *B. armeniaca* methanolic extract (BME) in positive mode; Figure S2: Base peak chromatogram (BPC) of Q-TOF LC/MS/MS analysis of *B. armeniaca* methanolic extract (BME) in negative mode; Figure S3: MS/MS spectrum of peak 2: L-Histidine; Figure S4: MS/MS spectrum of peak 7: L-Glutamic acid; Figure S5: MS/MS spectrum of peak 12: Caffeic acid; Figure S6: MS/MS spectrum of peak 16: Protocatechuic acid; Figure S7: MS/MS spectrum of peak 20: L-Pyroglutamic acid; Figure S8: MS/MS spectrum of peak 29: 4-Hydroxybenzoic acid; Figure S9: MS/MS spectrum of peak 36: Aesculin; Figure S10: MS/MS spectrum of peak 40: Isorhamnetin-3-*O*-glucoside; Figure S11: MS/MS spectrum of peak 44: Baicalein-7-*O*-glucuronide; Figure S12: MS/MS spectrum of peak 47: Kaempferol-3-*O*-robinoside-7-*O*-rhamnoside; Figure S13: MS/MS spectrum of peak 48: Cyanidin-3-*O*-rutinoside; Figure S14: MS/MS spectrum of peak 51: Luteolin-6-*C*-glucoside; Figure S15: MS/MS spectrum of peak 52: Diosmetin-7-*O*-rutinoside; Figure S16: MS/MS spectrum of peak 53: Quercetin-4′-*O*-glucoside; Figure S17: MS/MS spectrum of peak 54: Apigenin 8-*C*-glucoside; Figure S18: MS/MS spectrum of peak 57: Luteolin-3′, 7-di-*O*-glucoside; Figure S19: MS/MS spectrum of peak 58: Delphinidin 3-*O*-rutinoside; Figure S20: MS/MS spectrum of peak 61: Isosakuranetin-7-*O*-neohesperidoside; Figure S21: MS/MS spectrum of peak 63: Luteolin-8-*C*-glucoside; Figure S22: MS/MS spectrum of peak 65: Isorhamnetin-3-*O*-rutinoside; Figure S23: MS/MS spectrum of peak 76: Isorhamnetin; Figure S24: MS/MS spectrum of peak 79: Apigenin.
