# Supplementary material for: Novel Neuroprotective Potential of Bunchosia armeniaca (Cav.) DC against Lipopolysaccharide Induced Alzheimer’s Disease in Mice
Source: Plants (Basel). 2022 Jul 7;11(14):1792. doi: 10.3390/plants11141792 (PMC9322164; doi:10.3390/plants11141792)
Supplement: Supplementary file 1 [file plants-11-01792-s001.zip › plants-1763707-supplementary.pdf]

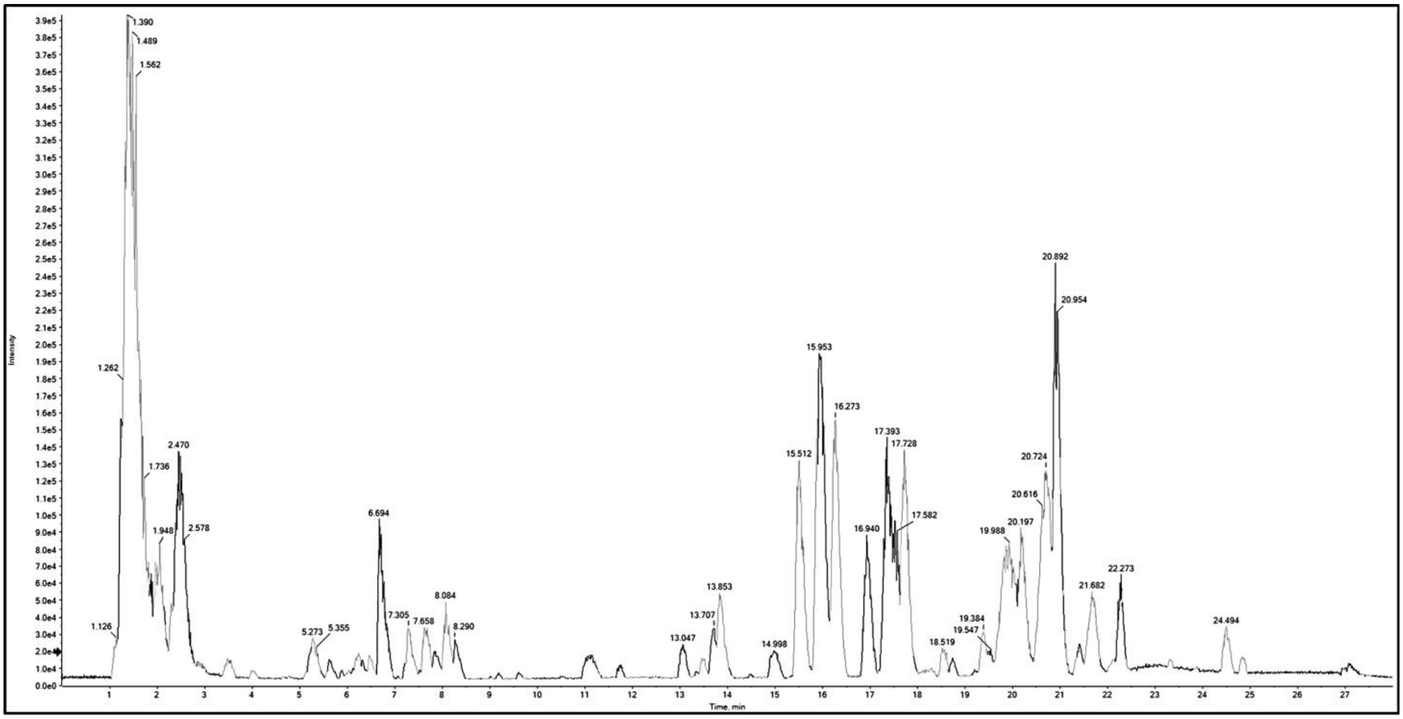

Figure S1. Base peak chromatogram (BPC) of Q-TOF LC/MS/MS analysis of *B. armeniaca* methanolic extract (BME) in positive mode.

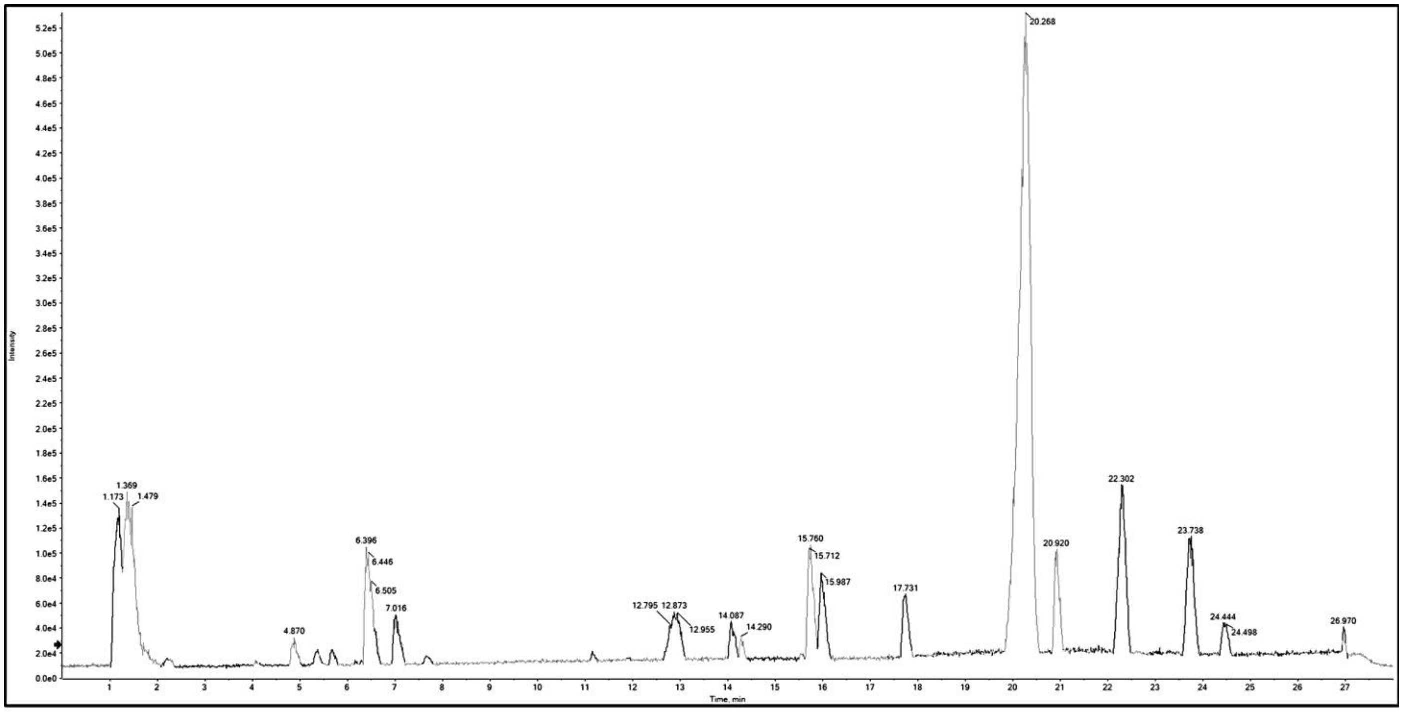

Figure S2. Base peak chromatogram (BPC) of Q-TOF LC/MS/MS analysis of *B. armeniaca* methanolic extract (BME) in negative mode.

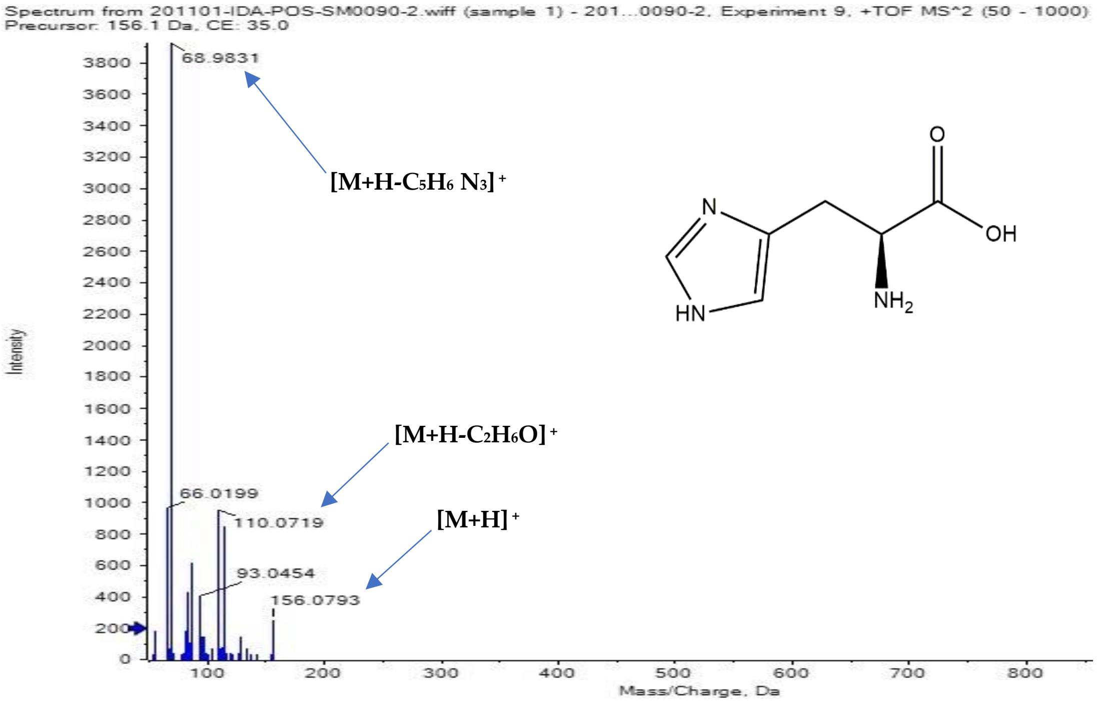

Figure S3. MS/MS spectrum of peak 2: L-Histidine.

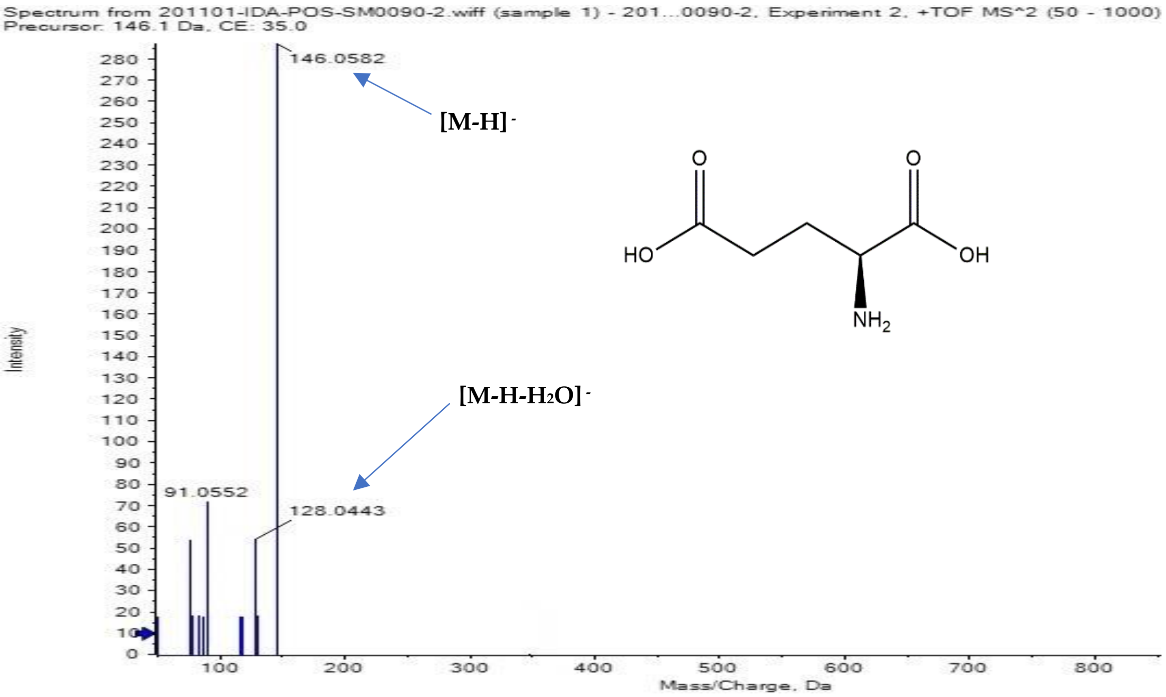

Figure S4. MS/MS spectrum of peak 7: L-Glutamic acid.

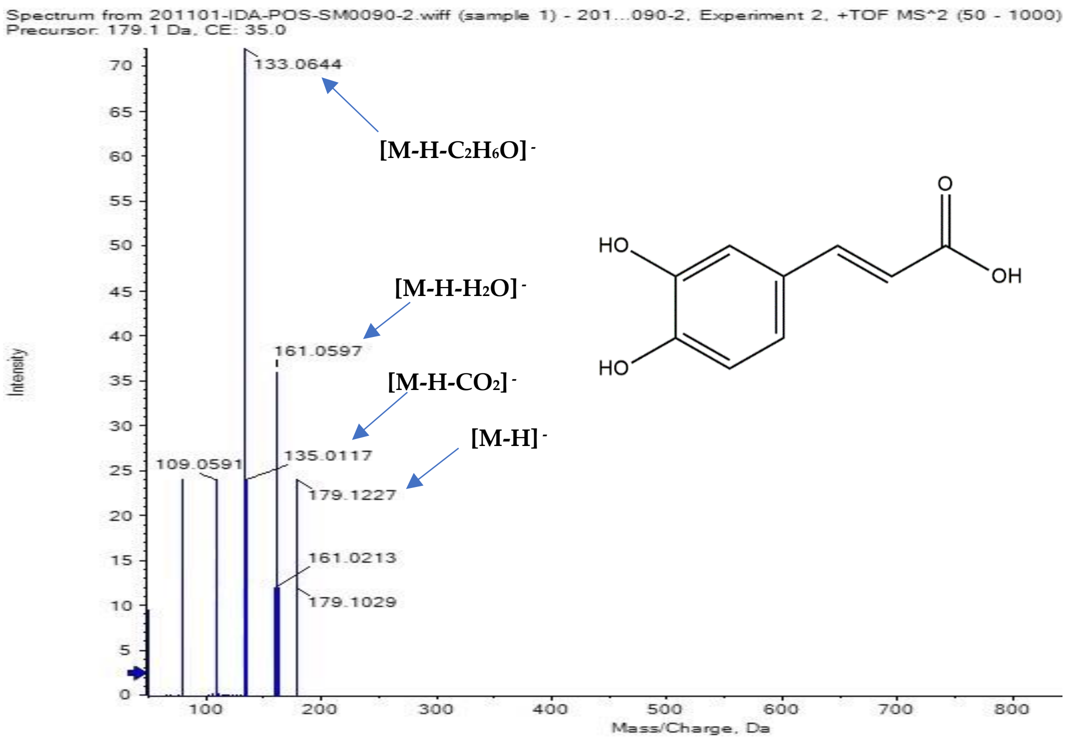

Figure S5. MS/MS spectrum of peak 12: Caffeic acid.

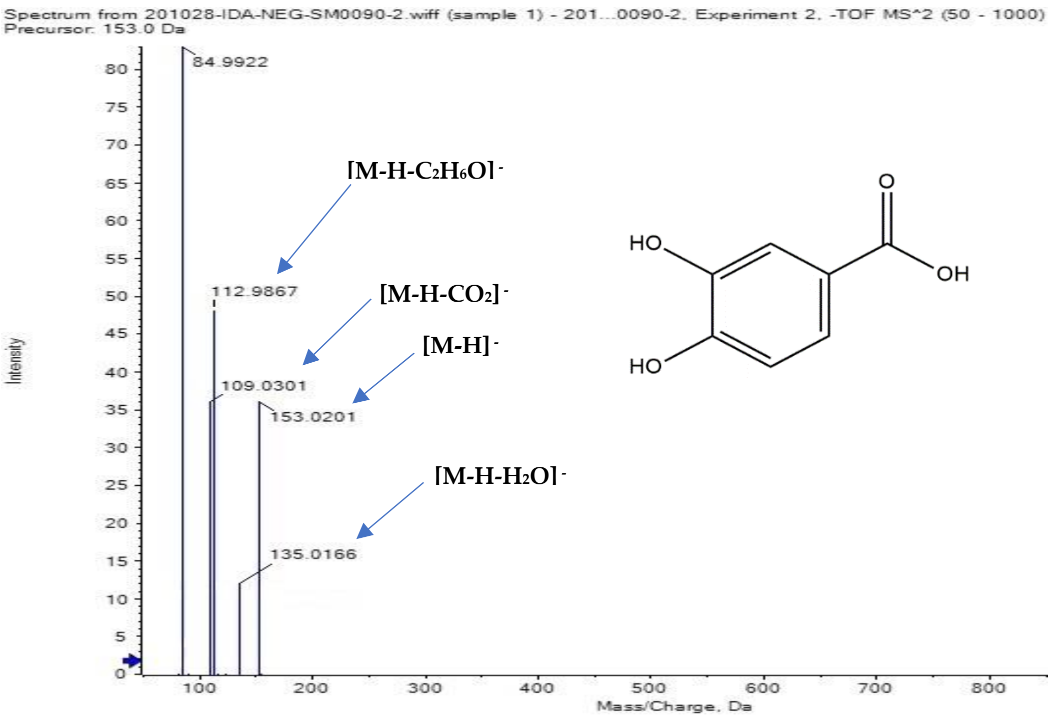

Figure S6. MS/MS spectrum of peak 16: Protocatechuic acid.

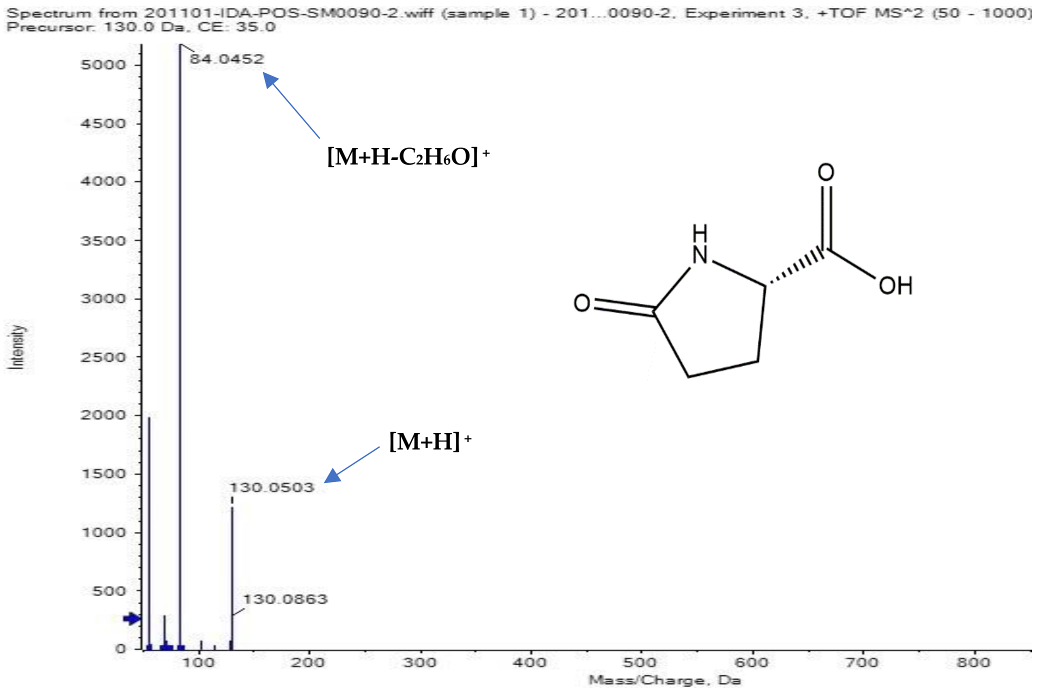

Figure S7. MS/MS spectrum of peak 20: L-Pyroglutamic acid.

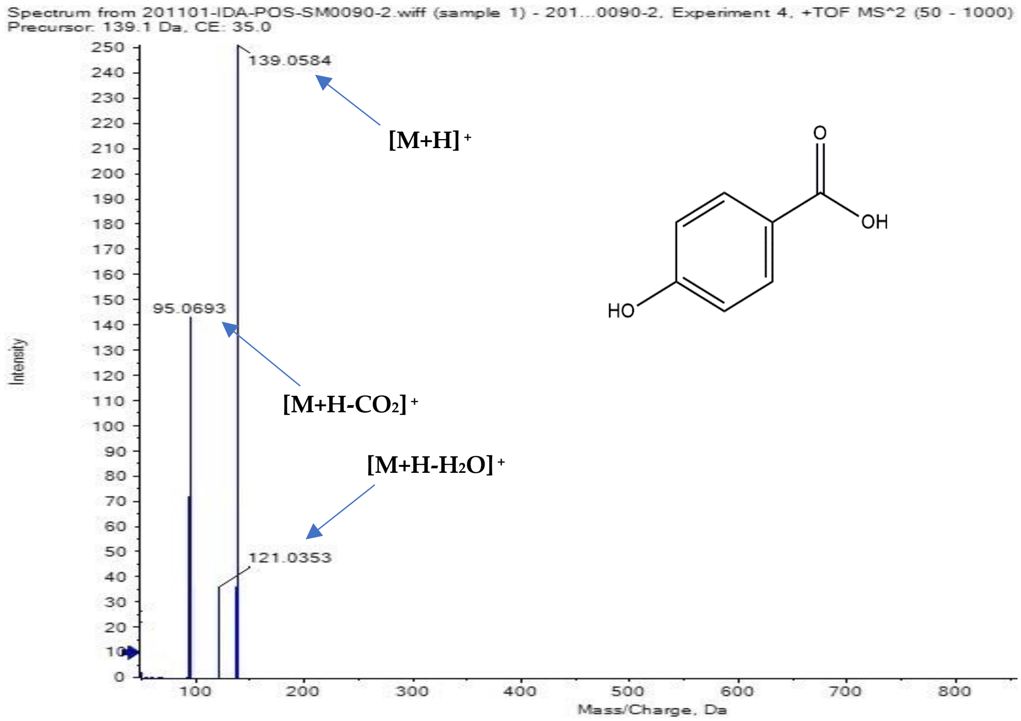

Figure S8. MS/MS spectrum of peak 29: 4-Hydroxybenzoic acid.

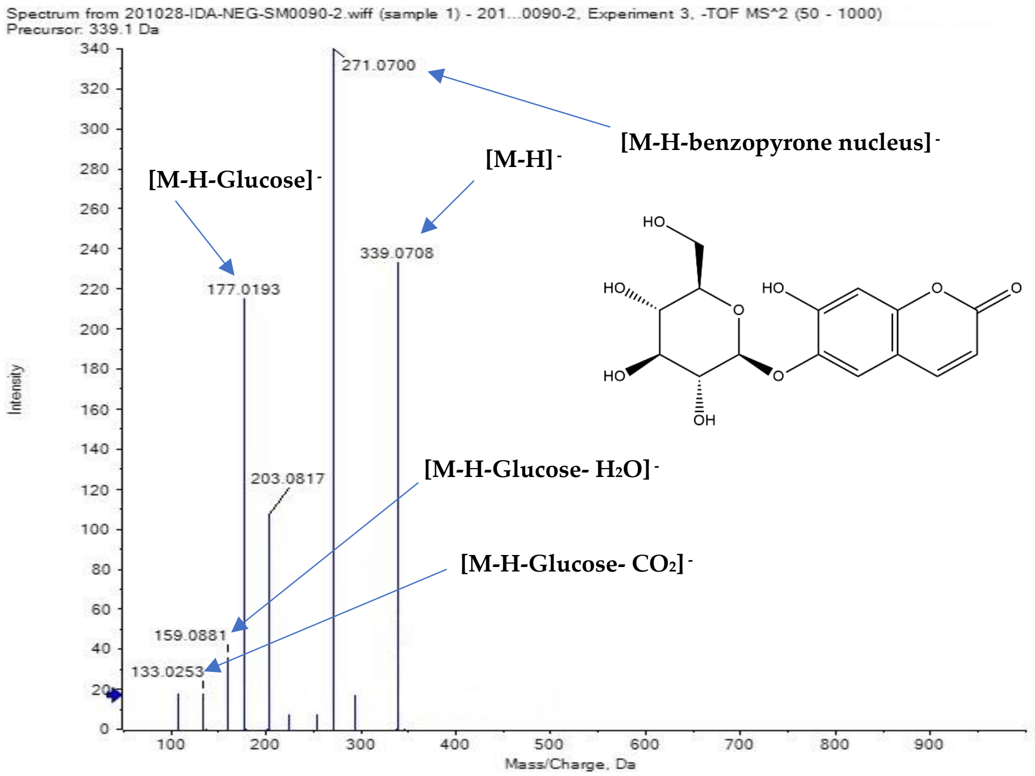

Figure S9. MS/MS spectrum of peak 36: Aesculin.

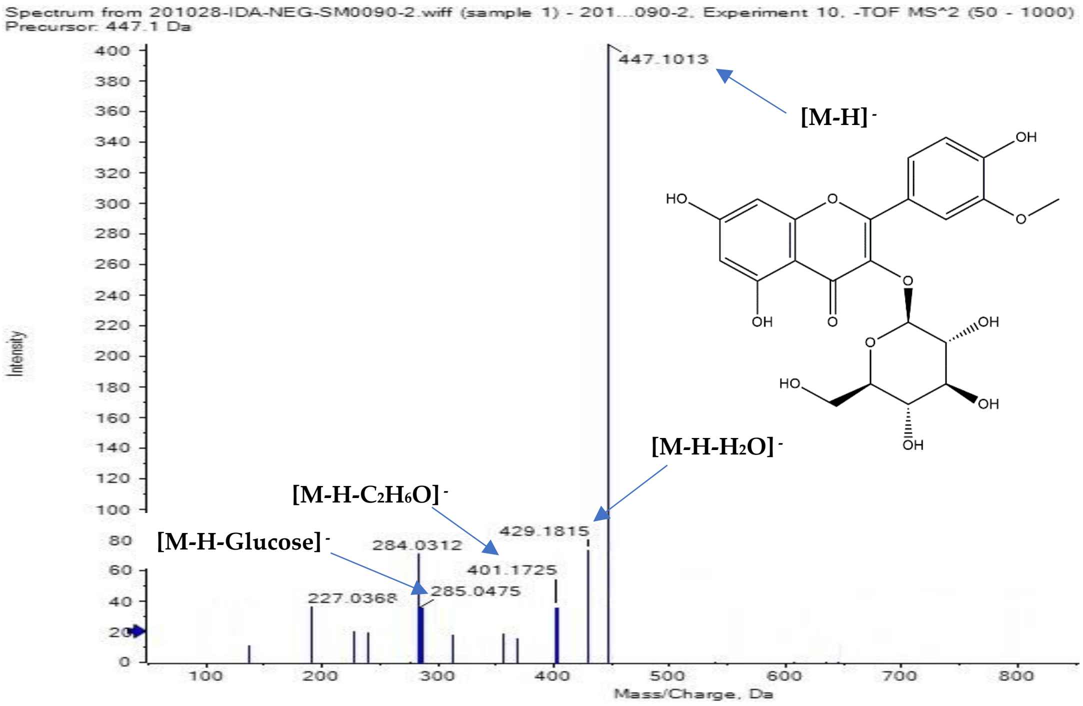

Figure S10. MS/MS spectrum of peak 40: Isorhamnetin-3-*O*-glucoside.

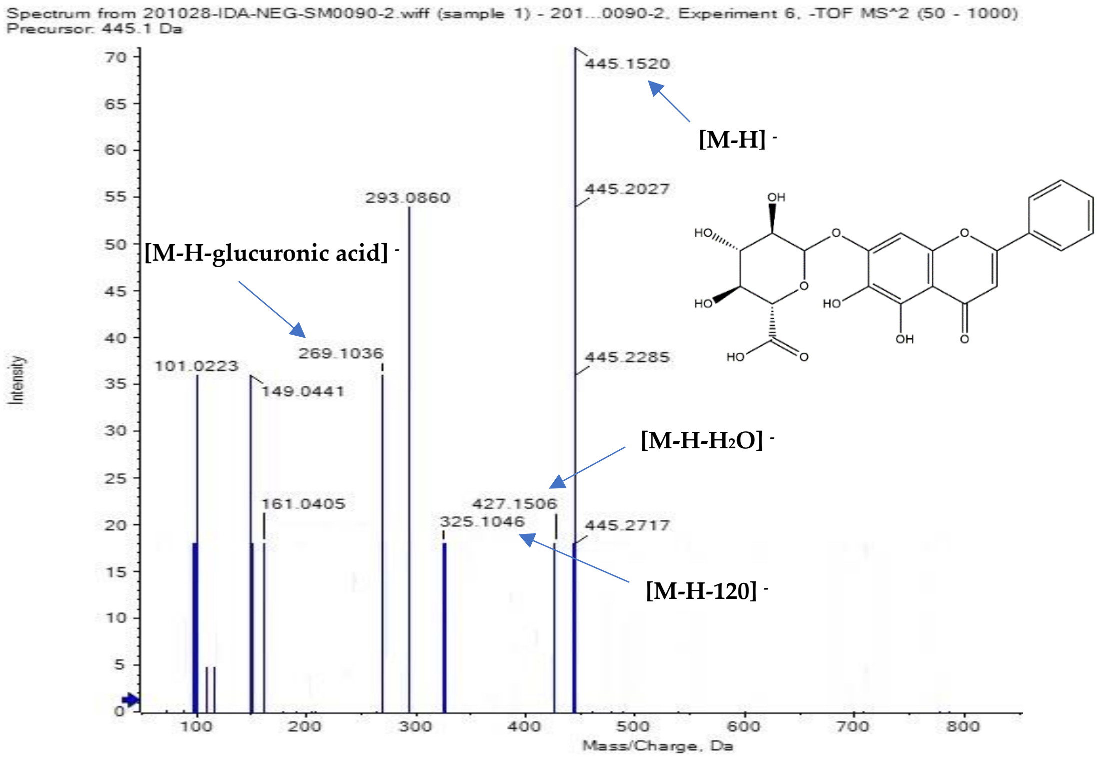

Figure S11. MS/MS spectrum of peak 44: Baicalein-7-*O*-glucuronide.

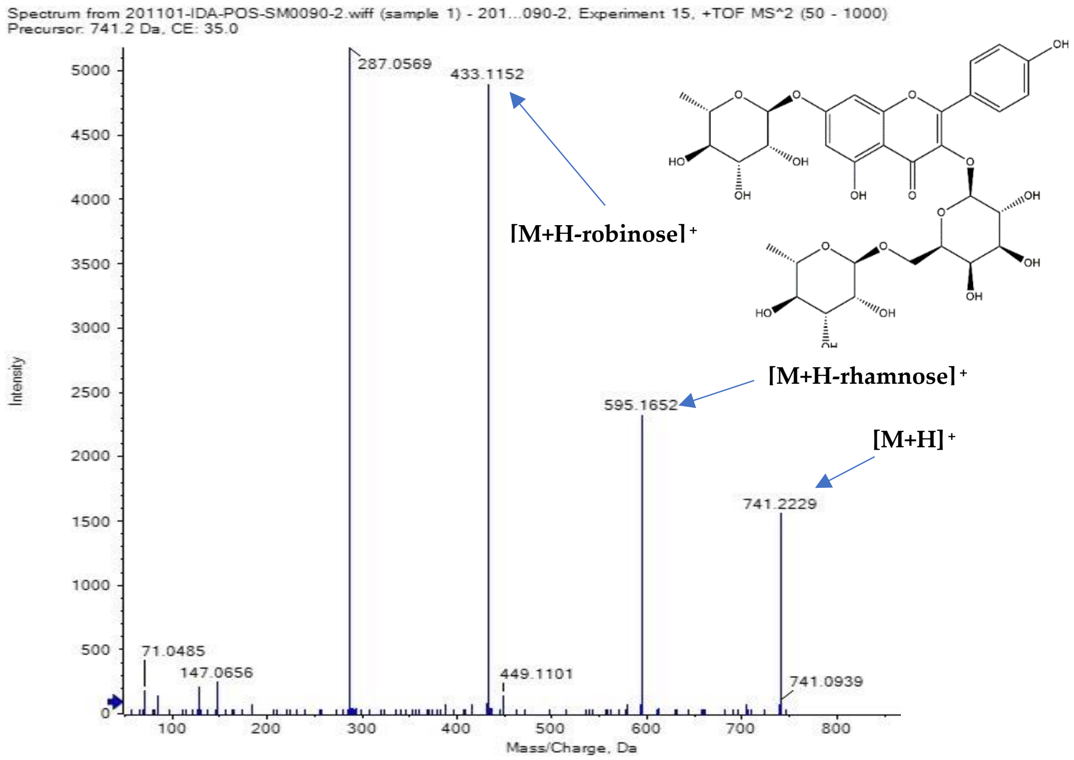

Figure S12. MS/MS spectrum of peak 47: Kaempferol-3-*O*-robinoside-7-*O*-rhamnoside.

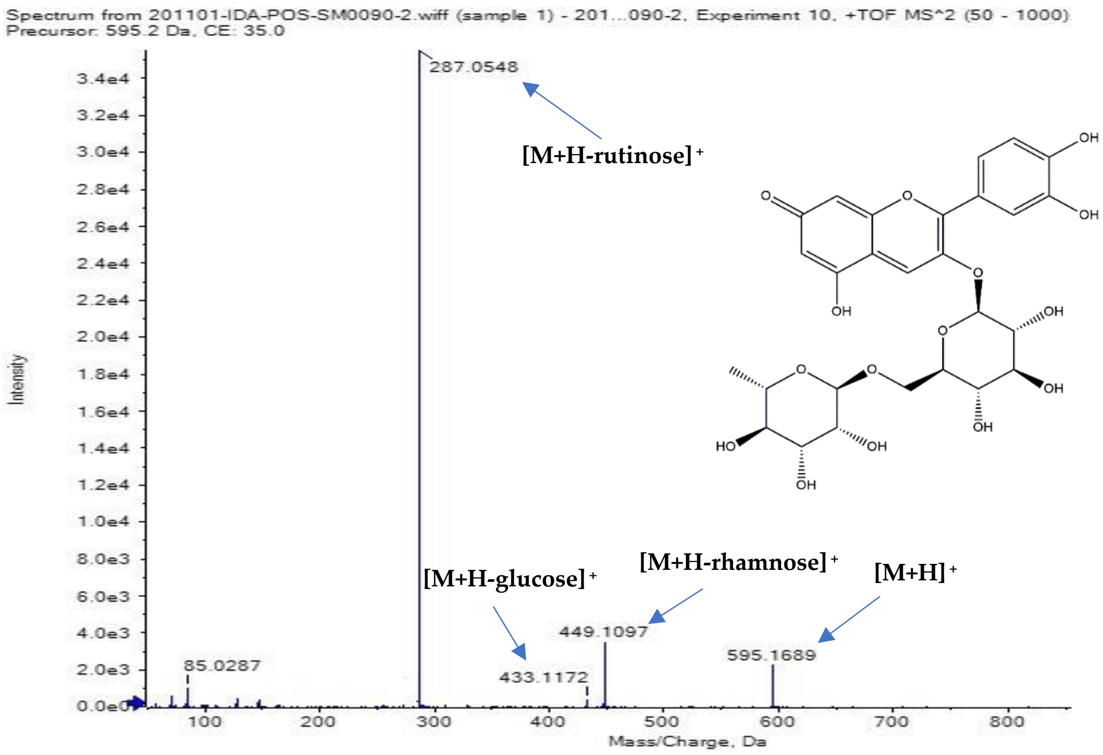

Figure S13. MS/MS spectrum of peak 48: Cyanidin-3-*O*-rutinoside.

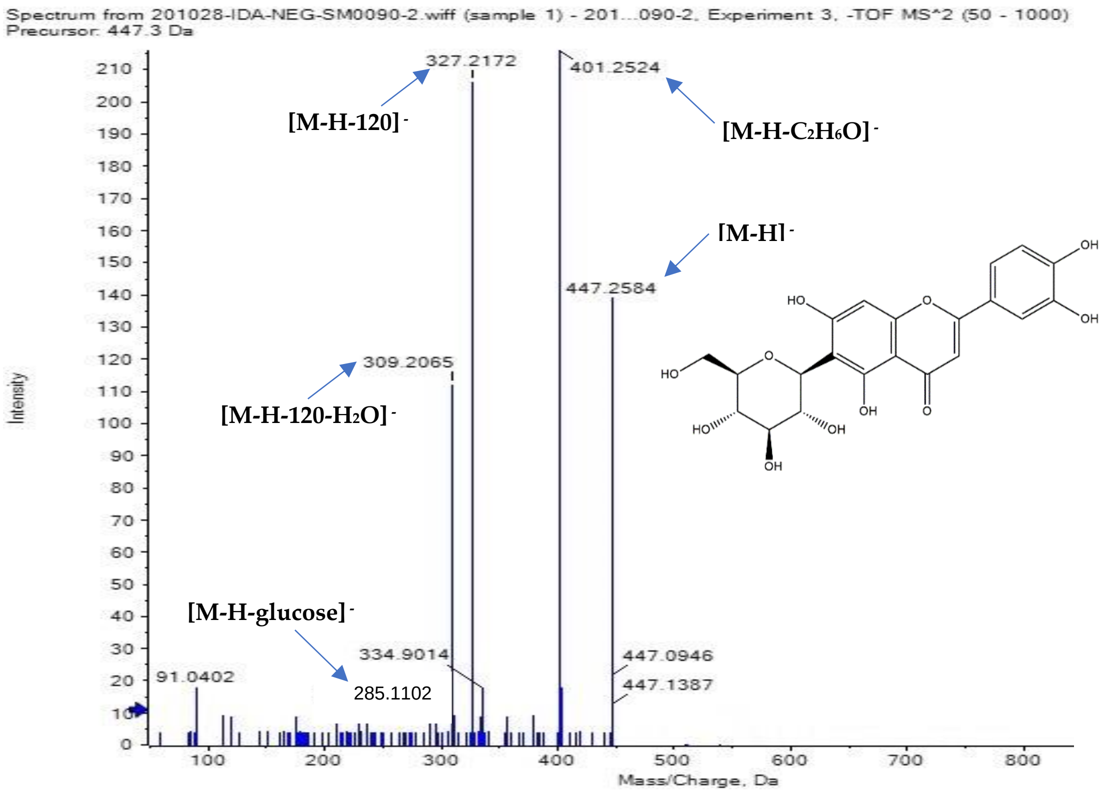

Figure S14. MS/MS spectrum of peak 51: Luteolin-6-C-glucoside.

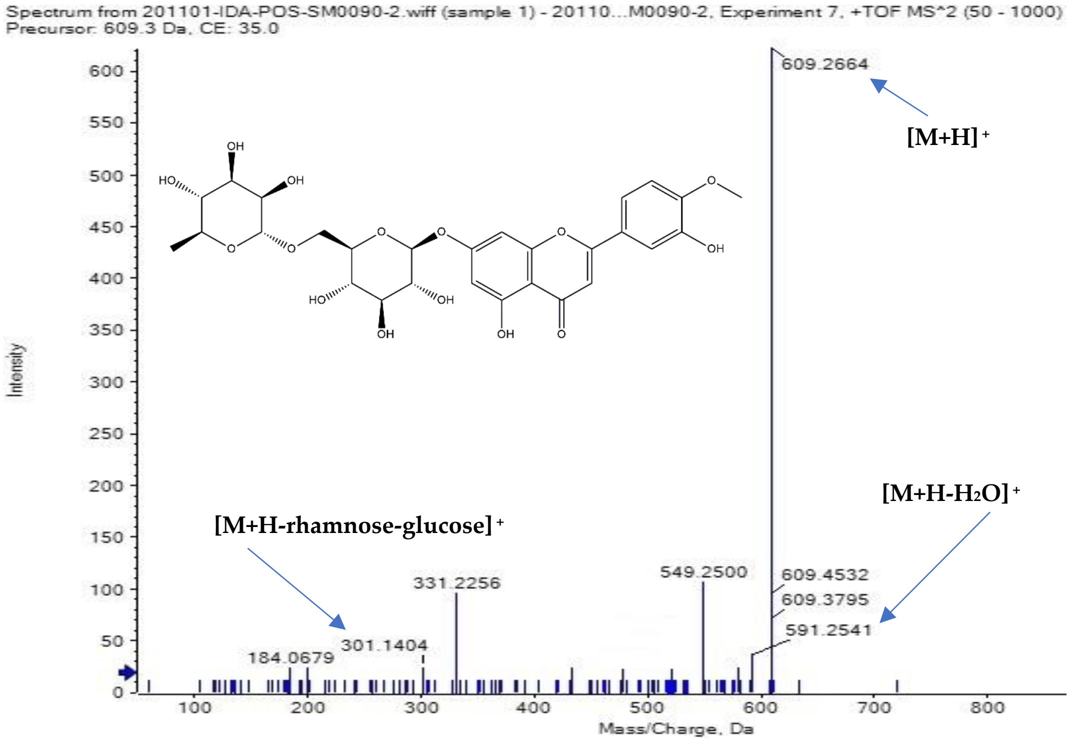

Figure S15. MS/MS spectrum of peak 52: Diosmetin -7-*O*-rutinoside.

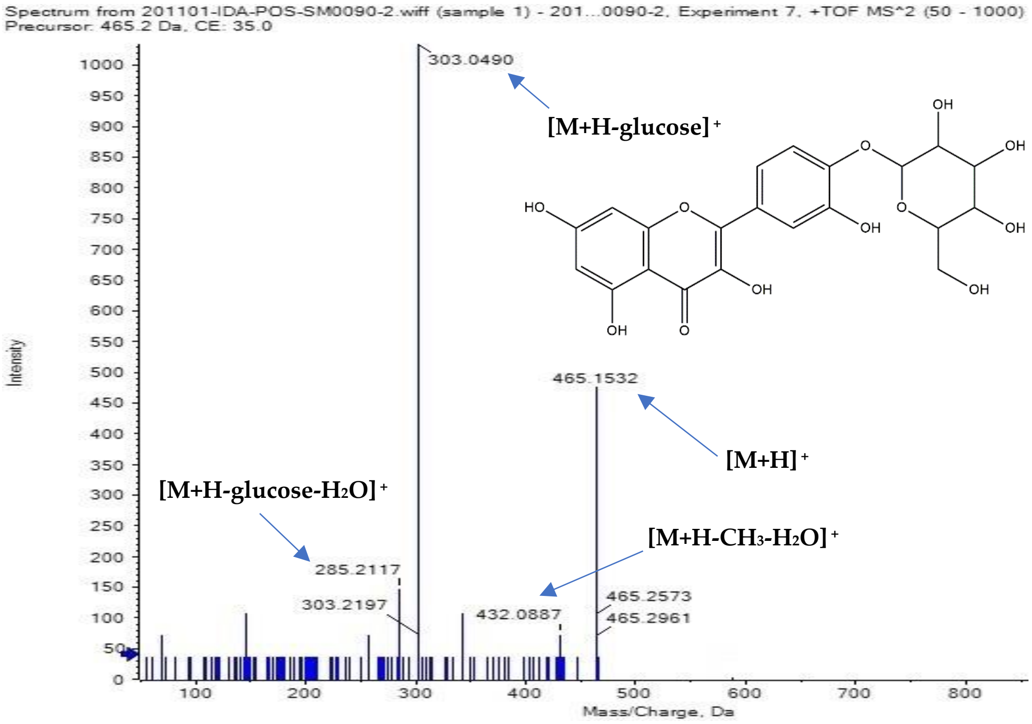

Figure S16. MS/MS spectrum of peak 53: Quercetin-4'-*O*-glucoside.

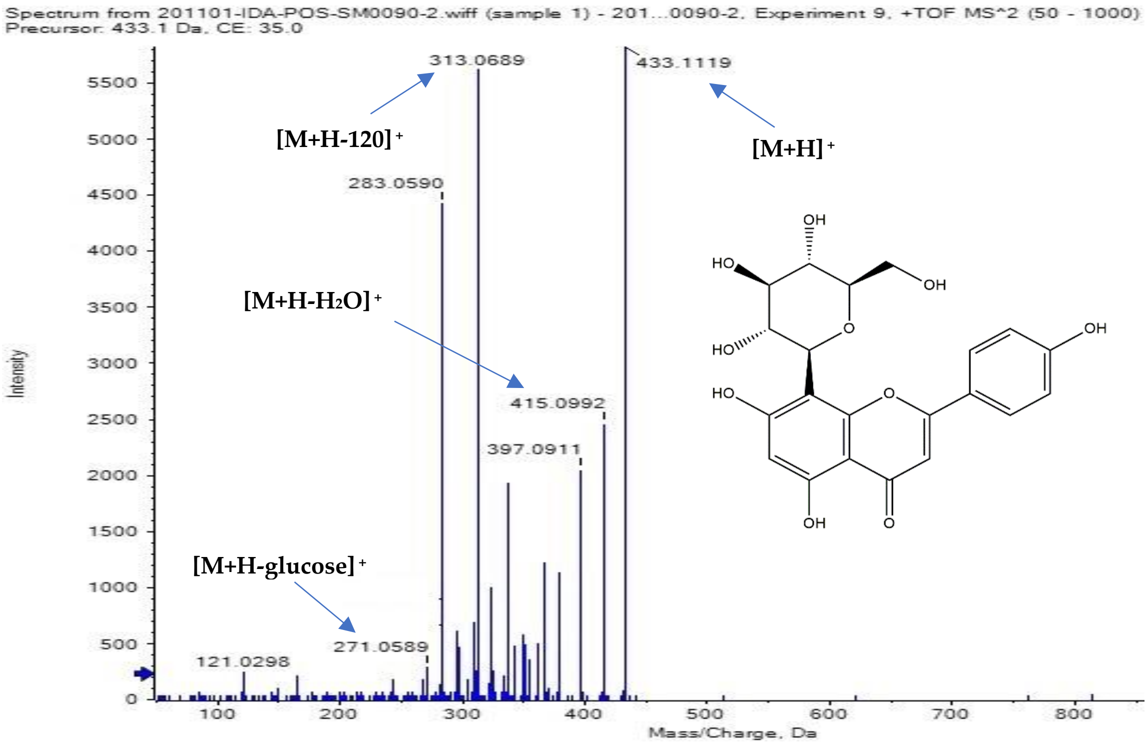

Figure S17. MS/MS spectrum of peak 54: Apigenin 8-*C*-glucoside.

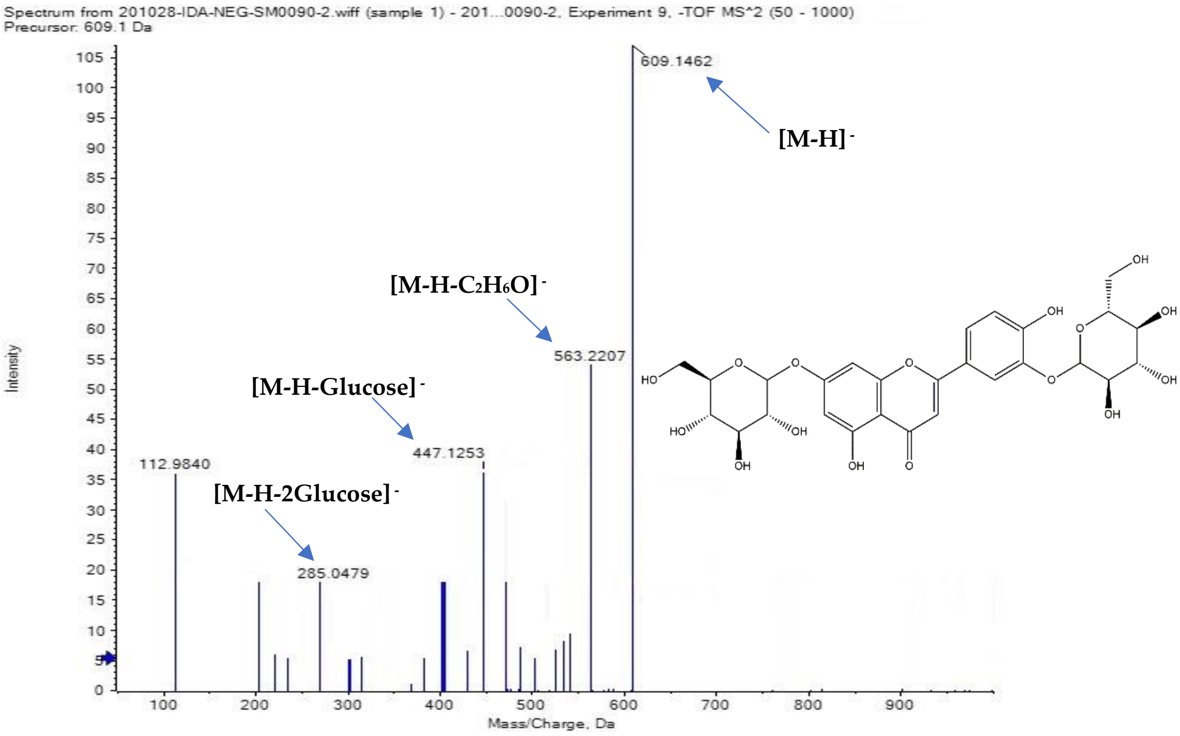

Figure S18. MS/MS spectrum of peak 57: Luteolin-3', 7-di-*O*-glucoside.

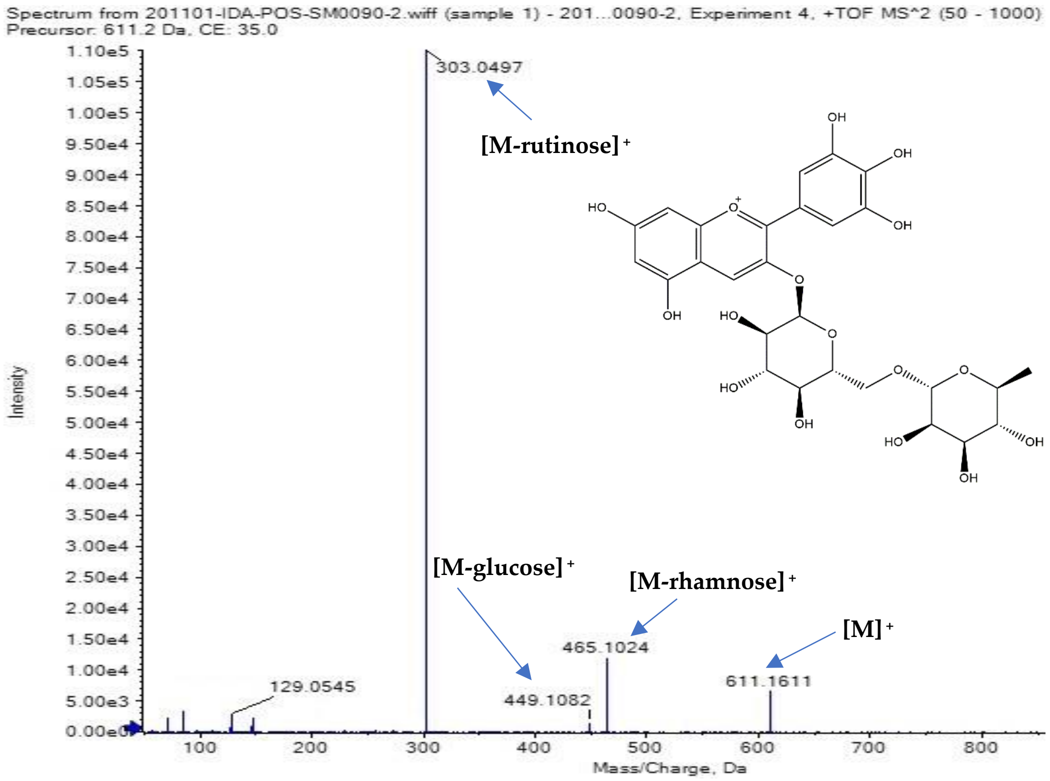

Figure S19. MS/MS spectrum of peak 58: Delphinidin 3-*O*-rutinoside.

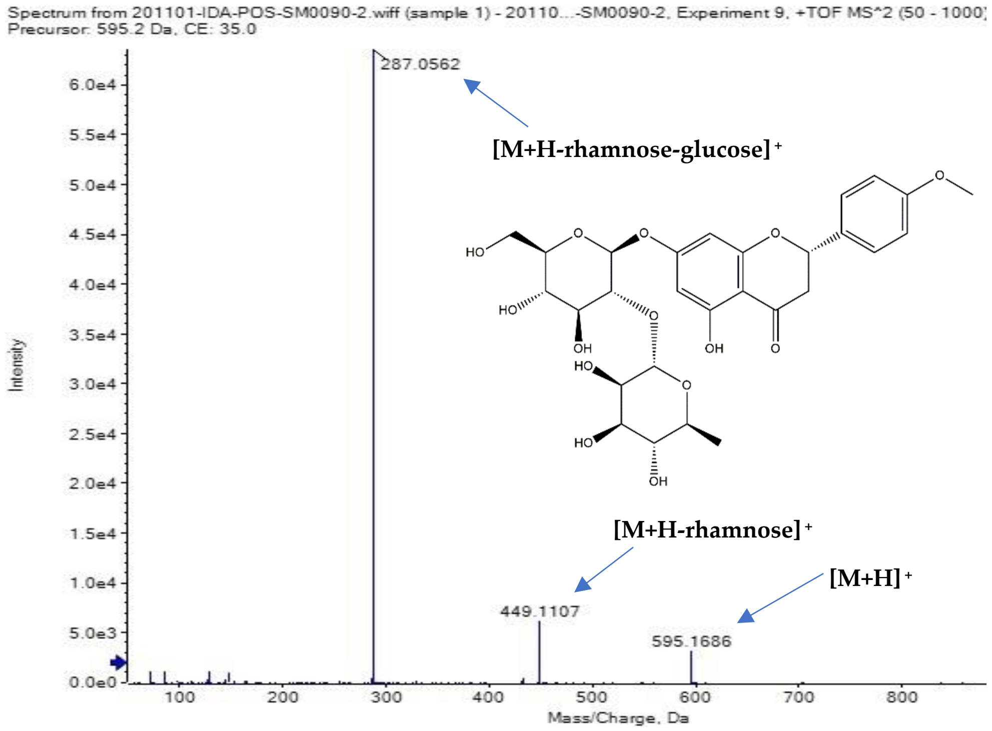

Figure S20. MS/MS spectrum of peak 61: Isosakuranetin-7-*O*-neohesperidoside.

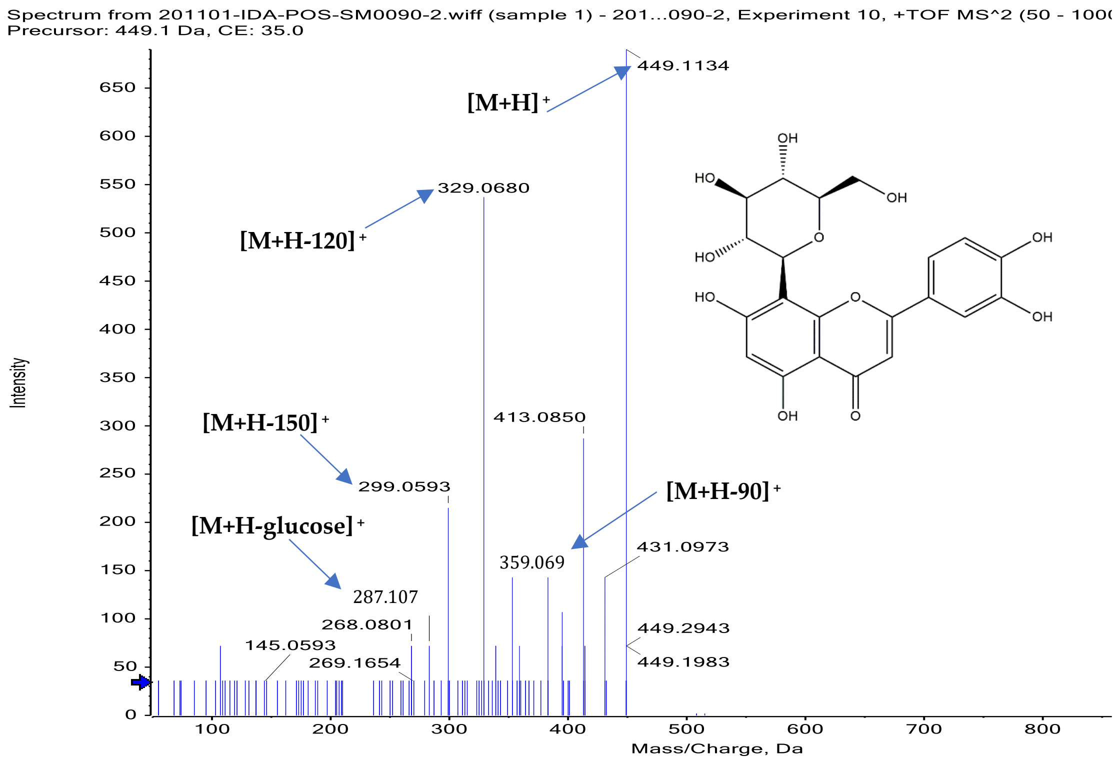

Figure S21. MS/MS spectrum of peak 63: Luteolin-8-*C*-glucoside.

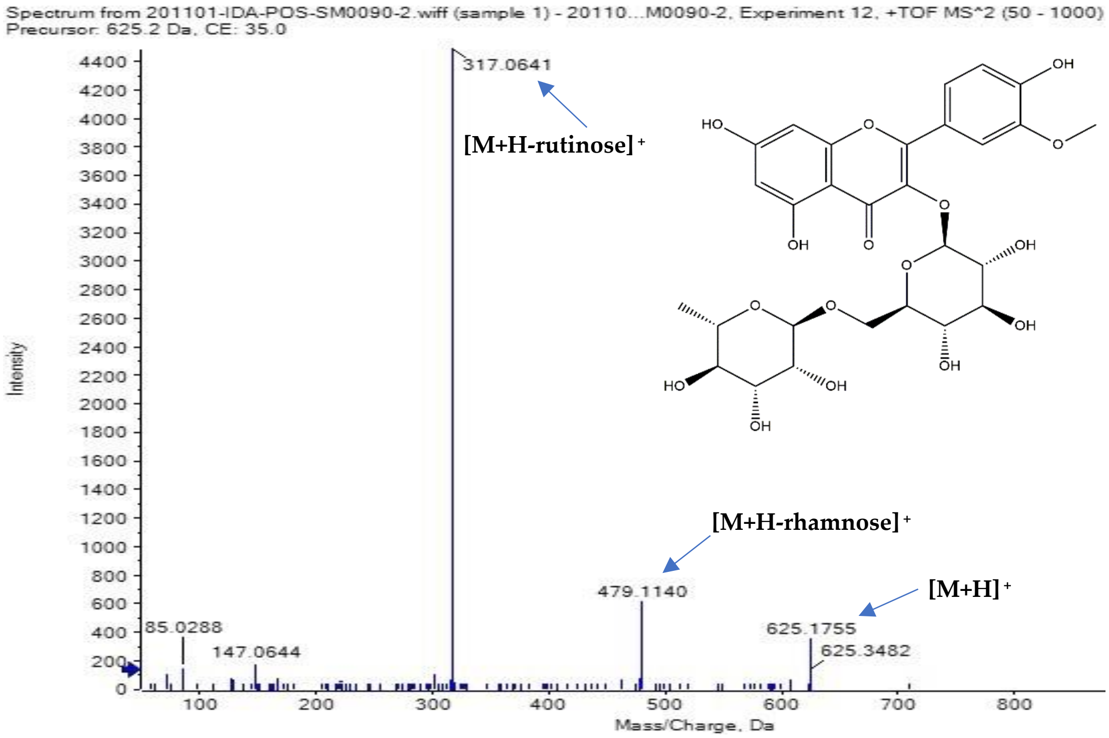

Figure S22. MS/MS spectrum of peak 65: Isorhamnetin-3-*O*-rutinoside.

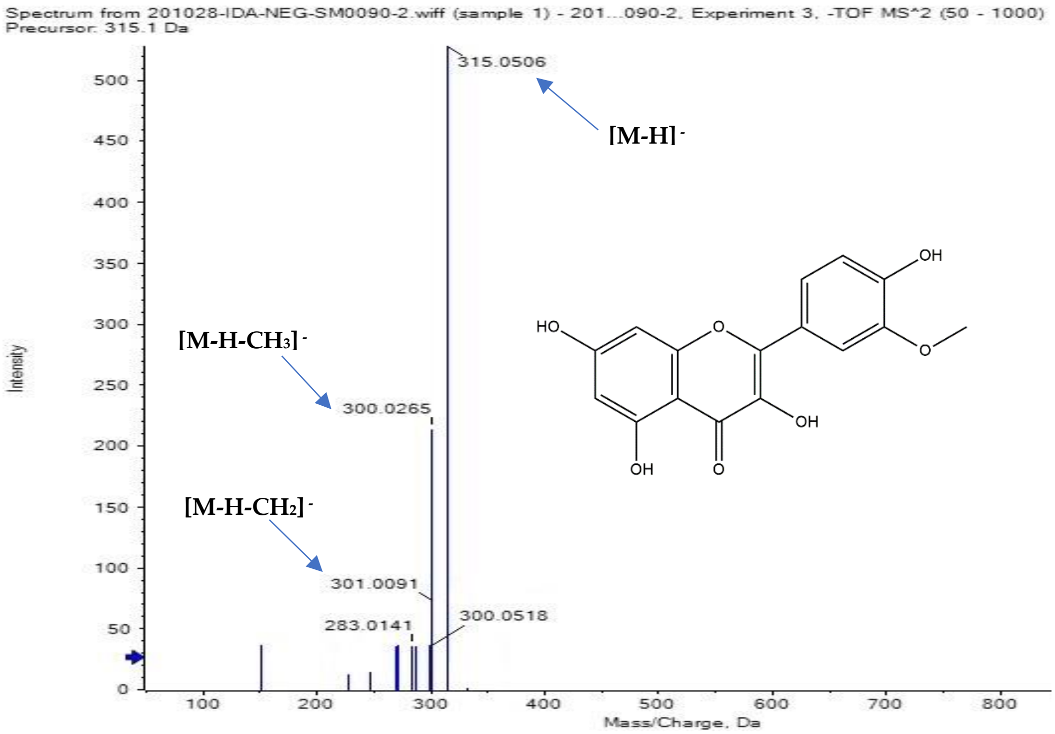

Figure S23. MS/MS spectrum of peak 76: Isorhamnetin.

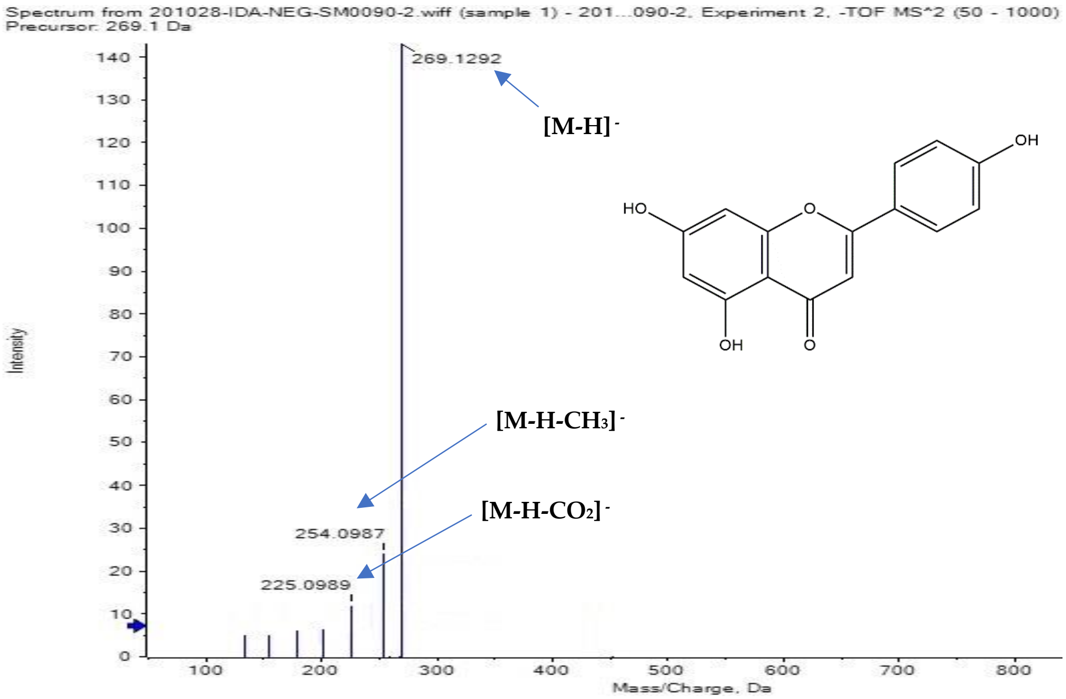

Figure S24. MS/MS spectrum of peak 79: Apigenin.
